# The Japanese encephalitis virus NS1 protein concentrates ER membranes in a cytoskeleton-independent manner to facilitate viral replication

**DOI:** 10.1128/jvi.02113-24

**Published:** 2025-02-05

**Authors:** Shengda Xie, Xinxin Lin, Qing Yang, Miaolei Shi, Xingmiao Yang, Ziyu Cao, Ruibing Cao

**Affiliations:** 1 MOE Joint International Research Laboratory of Animal Health and Food Safety, College of Veterinary Medicine, Nanjing Agricultural University 261674 , Nanjing, China; University of North Carolina at Chapel Hill, Chapel Hill, North Carolina, USA

**Keywords:** Japanese encephalitis virus, NS1, main replication organelles, ER membranes, concentration, viral replication

## Abstract

Orthoflaviviruses remodel the endoplasmic reticulum (ER) network to construct replication organelles (ROs) for RNA replication. In this study, we demonstrate that the Japanese encephalitis virus (JEV) NS1 protein concentrates ER membranes in the perinuclear region, which provides a substantial membrane source for viral replication. Subsequently, the virus forms main replication organelles within this membrane-concentrated area to facilitate efficient replication. This process relies on the ER localization signal, glycosylation, dimerization, and membrane-binding sites of the NS1 protein. In conclusion, our study highlights the role of the NS1 protein in the formation of the ROs by JEV, providing new insights into orthoflavivirus replication.

**IMPORTANCE:**

Orthoflaviviruses use the endoplasmic reticulum (ER) membranes for replication by forming invaginations to assemble the replication organelles. Here, we found that Japanese encephalitis virus (JEV) utilizes the NS1 protein to concentrate a significant number of ER membranes in the perinuclear area, thereby providing a membrane source for viral replication and facilitating the formation of main replication organelles (MROs). This process depends on the ER localization signals of NS1, as well as its glycosylation, dimerization, and membrane-binding sites, but not on the cytoskeleton. In summary, our study highlights how NS1 remodels ER membranes to facilitate the formation of MROs for JEV, thereby accelerating viral replication.

## INTRODUCTION

Japanese encephalitis (JE) is caused by the Japanese encephalitis virus (JEV), an orthoflavivirus transmitted through mosquito bites. JEV was first isolated from the brain of a person who died from encephalitis in Japan in 1934 (1). Although JE incidence remains uncertain due to limited surveillance in many endemic countries, JEV is considered to be the leading cause of viral encephalitis in Asia (2). It is estimated that there are between 67,900 and 100,000 cases of JE annually, with 75% of these cases occurring in children <14 years old (2, 3). As an arthropod-borne virus, JEV transmits between mosquito-borne and vertebrate hosts, especially pigs and wading birds (4). Humans are the dead-end hosts for JEV, and pigs are the amplified hosts for this virus (5). Virus replication is essential for the spread of the virus; therefore, studying the replication of JEV is critical for effective disease prevention and control.

JEV primarily replicates at the endoplasmic reticulum (ER) and forms replication organelles (ROs) to facilitate viral genome replication (6, 7). During this process, there are significant rearrangements of ER membranes leading to the formation of various structures like vesicle packets (VP) and convoluted membranes to support virus replication (7, 8). Nonstructural proteins, such as NS1, NS2A, NS2B, NS4A, and NS4B, play important roles in remodeling the ER for replication organelle formation (7, 9–12). A recent study has demonstrated that orthoflaviviruses can concentrate ER membranes in the perinuclear replication region to enhance replication efficiency by promoting ER clustering, growth, merging, directional movement, and convergence (13). However, the mechanism by which orthoflaviviruses remodel ER membranes to mediate the formation of ROs remains unknown.

In this study, we found that JEV NS1 protein embeds into and concentrates the ER membranes through its hydrophobic amino acid sites to provide a membrane source for viral replication and facilitate the formation of the main replication organelles (MROs) for the virus.

## RESULTS

### JEV infection concentrates ER membranes

**Fig 1:**
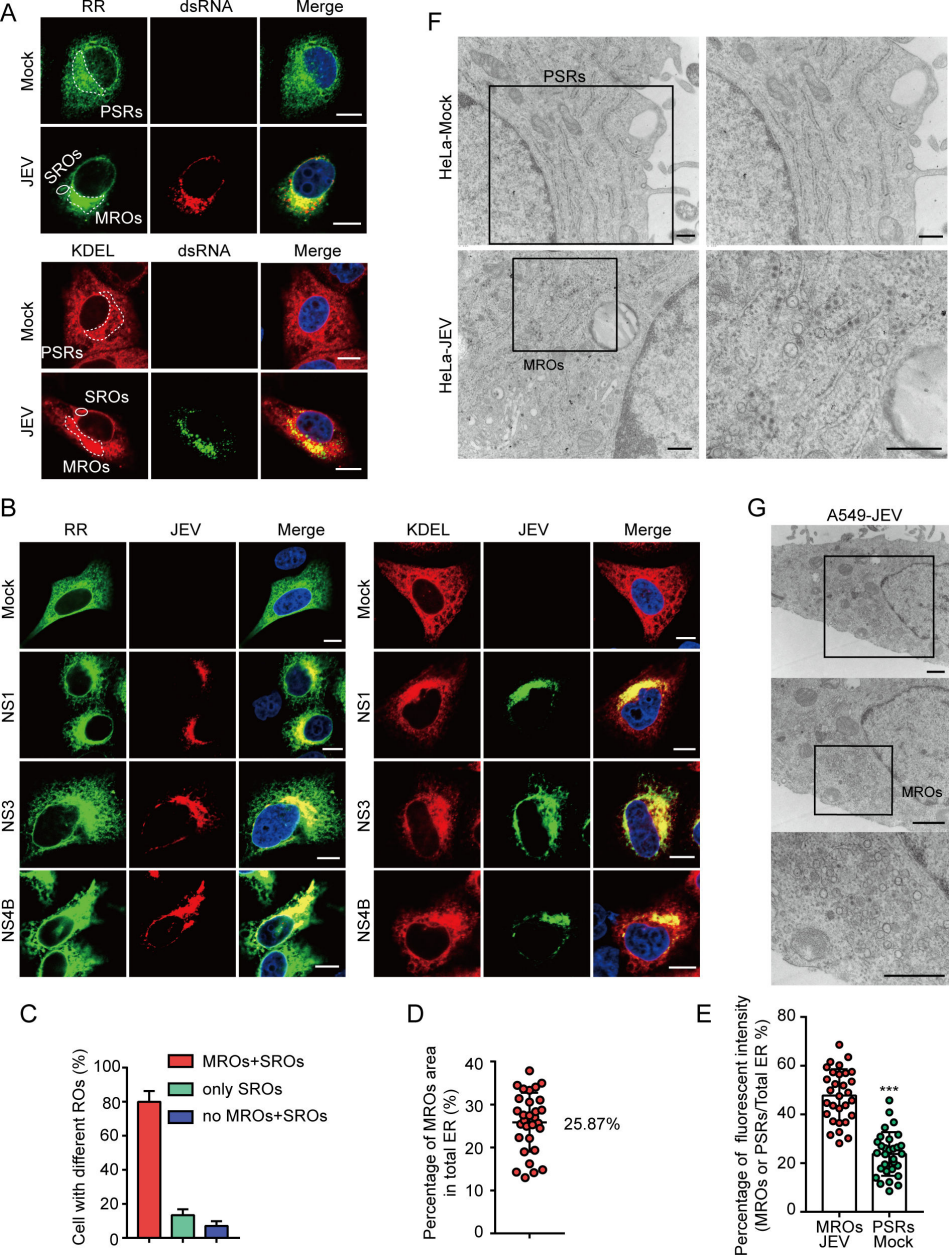
Concentrated ER membranes in JEV-infected cells. (**A and B**) HeLa cells were transfected with the RR or KDEL plasmids and then infected with the JEV NJ2008 strain at a multiplicity of infection (MOI) of 0.5, 36 hpi. Viral dsRNA (**A**), NS1 (**B**), NS3 (B), and NS4B (**B**) were stained with respective antibodies. Scale bars, 10 µm. Dotted circles in JEV-infected cells represent MROs (**A**). Solid circles in JEV-infected cells represent SROs (**A**). Dotted circles in mock cells represent PSRs (**A**). (**C**) The percentage of JEV-infected wild-type (WT) HeLa cells with three different types of ER clusters. Average of three counts, each with more than 40 cells. (**D**) Area percentage of MROs to the total ER. Each point represents a single cell from two independent experiments. (**E**) The percentage of ER fluorescence intensity in MROs or PSRs. Each point represents a single cell from two independent experiments, two-tailed *t*-test, ***P < 0.001. (**F**) TEM images of mock and JEV-infected (MOI = 0.5, 36 hpi) HeLa cells. Scale bars, 400 nm. (**G**) TEM images of JEV-infected A549 cells at 36 hpi (MOI = 0.5). Scale bars, 600 nm.

To investigate the remodeling of the ER membranes induced by JEV replication, mCherry-KDEL (KDEL) and RR-mNeonGreen (RR) plasmids were used for ER labeling. The KDEL sequence serves as an endoplasmic reticulum-resident signal, which is widely utilized for labeling ER membranes (14). In contrast, RR localizes to the ER via a transmembrane region of VAMP2 and an N-terminal arginine-rich sequence, which functions as an ER retention signal, representing a newly developed tool for ER membrane labeling (Fig. S1A and B) (15). HeLa cells were transfected with KDEL or RR plasmids and subsequently infected with the JEV NJ2008 strain. Confocal results indicated that JEV-infected cells exhibited multiple aggregated ER clusters located in the perinuclear region (PN), which were highly co-localized with viral double-stranded RNA (dsRNA, a marker of ROs; Fig. 1A). These findings suggest that ER membranes accumulate near viral ROs. To further confirm the association of these ER clusters with JEV ROs, JEV-infected cells were stained with antibodies against the viral NS1, NS3, and NS4B proteins, which are localized to the viral ROs (7, 16). Confocal results demonstrate that NS1, NS3, and NS4B proteins co-localized with ER clusters (Fig. 1B). To more precisely characterize ER clusters, we categorized JEV-infected cells into three groups (see Materials and Methods for categorization): (i) large concentrated areas of ER clusters referred to as viral MROs (indicated by dotted circles in JEV-infected cells in Fig. 1A); (ii) small concentrated areas of ER clusters termed viral small replication organelles (SROs, indicated by solid circles in JEV-infected cells in Fig. 1A); and (iii) regions lacking ER clusters, designated as no MROs and SROs. Approximately 93% of JEV-infected cells exhibited aggregated ER clusters, with MROs and SROs coexisting in 80% of cells, while only SROs were present in 13% of cells (Fig. 1C). In JEV-infected cells, MROs comprised 12.98%–37.84% (average value, 25.87%) of the total ER membrane area (Fig. 1D). In mock cells, we manually labeled regions resembling MROs and designated them as perinuclear similar regions (PSRs, indicated by dotted circles in mock cells in Fig. 1A). Compared to PSRs, MROs exhibit a greater concentration of ER membranes (Fig. 1E). Next, transmission electron microscopy (TEM) was used to observe the ultrastructure of JEV MROs and PSRs. A continuous network of ER membranes was observed in PSRs, while MROs exhibited the characteristic VP structure (Fig. 1F and G). These results suggest that JEV infection concentrates ER membranes to form MROs.

**Fig 2:**
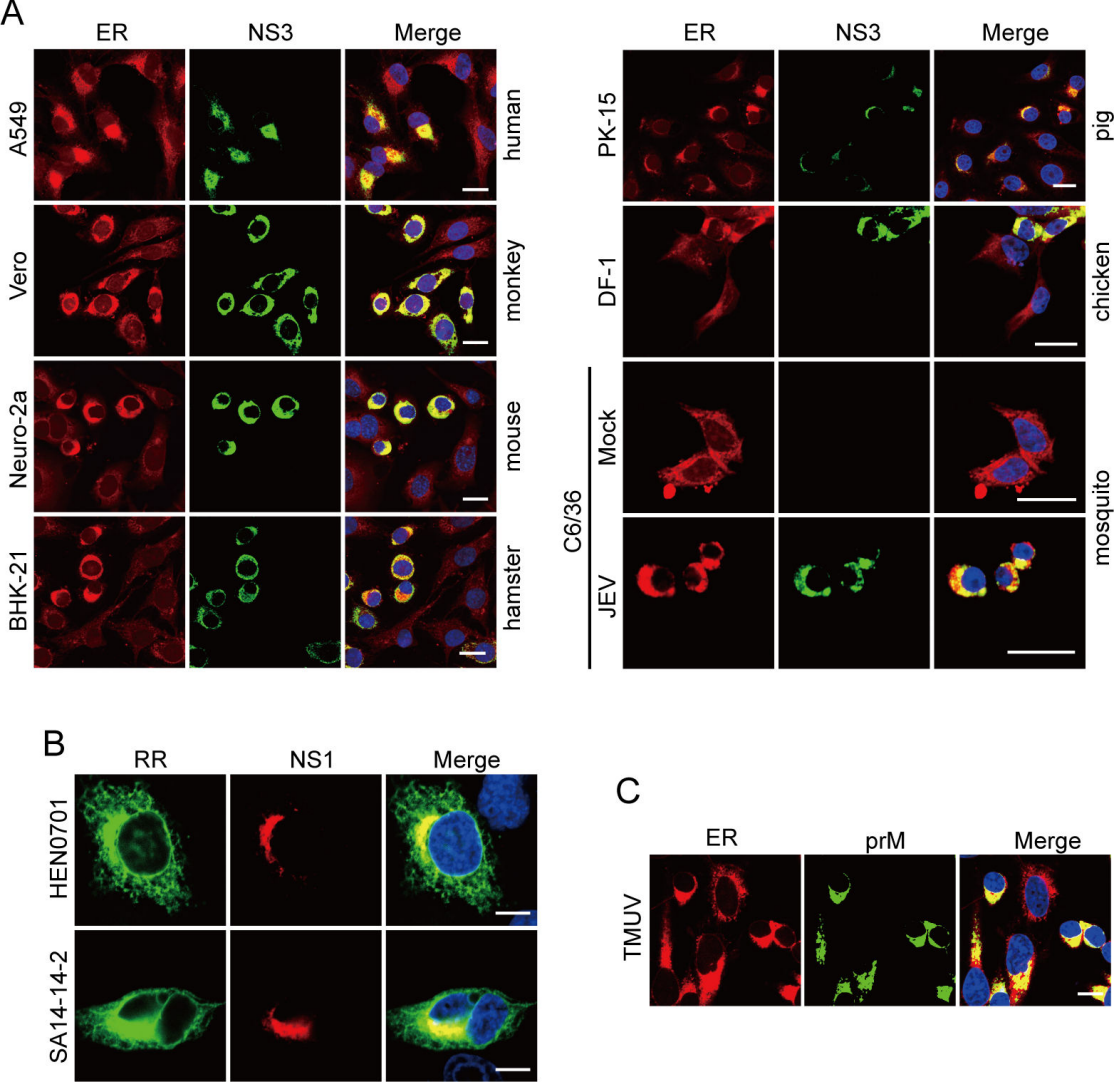
JEV concentrates ER membranes in different cell types. (**A**) Different cells types were infected with the JEV NJ2008 strain at an MOI of 0.5 (Vero, PK-15, and BHK-21 cells for 24 hpi; A549, Neuro-2a, and DF-1 cells for 36 hpi; C6/36 cells for 48 hpi). JEV infection in the ER was observed using the confocal microscope. The ER was stained with dye. Scale bars, 20 µm. (**B**) HeLa cells were infected with JEV HEN0701 and SA14-14-2 strains at an MOI of 0.5 for 36 hpi. The concentration of ER membranes was observed using the confocal microscope. Scale bars, 10 µm. (**C**) BHK-21 cells were infected with the TMUV XZ2012 strain at an MOI of 1 for 24 hpi. The concentration of ER membranes was observed using the confocal microscope. The ER was stained with dye. Scale bars, 10 µm.

JEV is a multi-host virus that infects a diverse range of animals, including mammals, birds, and mosquitoes. Consequently, we investigated whether JEV induces the ER membrane concentration in various host cells. JEV can concentrate ER membranes in multiple host cell types (Fig. 2A). In addition to NJ2008 strain (GIII), both HEN0701 strain (GI) and SA14-14-2 strain (vaccine strain) are capable of forming MROs (Fig. 2B). Notably, Tembusu virus (TMUV), another orthoflavivirus causing severe egg-drop and encephalitis in domestic waterfowl, also concentrates ER membranes in the perinuclear region (Fig. 2C). This observation suggests that such membrane concentration may be a common phenomenon in orthoflavivirus infections.

### JEV NS1 protein concentrates ER membranes

**Fig 3:**
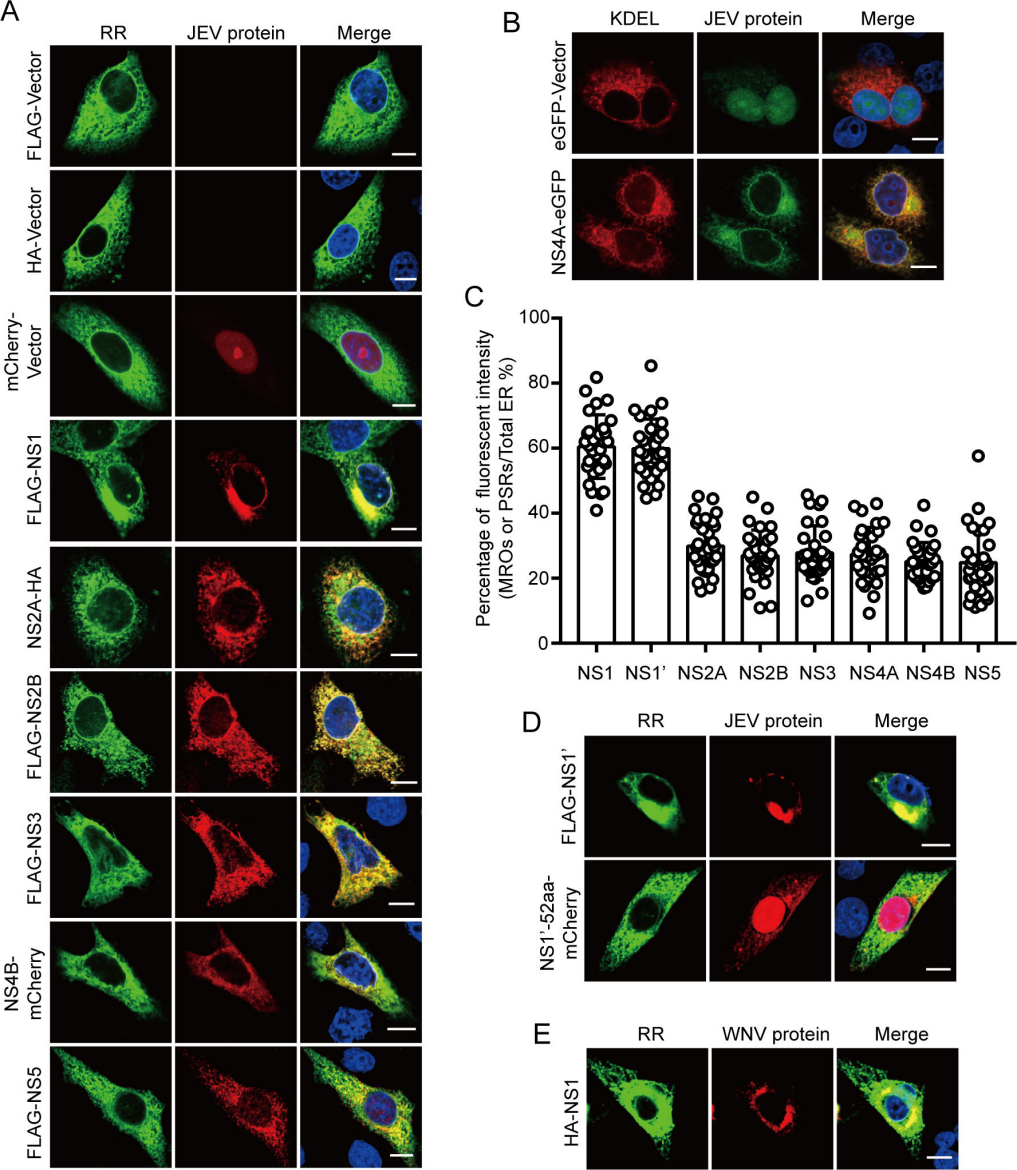
JEV NS1 and NS1’ proteins mediate membrane concentration. (**A and B**) HeLa cells were co-transfected with plasmids encoding either RR or KDEL, along with individual JEV non-structural proteins. The concentration of ER membranes was observed using the confocal microscope. The FLAG-tagged plasmids expressing JEV NS1, NS2B, NS3, and NS5 proteins. The mCherry-tagged plasmid expressing JEV NS4B protein. The HA-tagged plasmids expressing JEV NS2A (**A**). The eGFP-tagged plasmid expressing JEV NS4A protein (**B**). Scale bars, 10 µm. (**C**) Quantification of ER membrane concentration shown in A and B. Each point represents a single cell from two independent experiments. The statistics for WT NS1 are the same set as used in Fig. 4C. (**D and E**) HeLa cells were co-transfected with the RR and the JEV NS1' plasmid (D top), the mCherry-tagged plasmid expressing 52 shifted amino acids (D bottom), or the WNV NS1 plasmid (**E**). The concentration of ER membranes was observed using the confocal microscope. Scale bars, 10 µm.

To investigate the mechanism by which JEV concentrates ER membranes, we transfected plasmids encoding JEV non-structural proteins individually into HeLa cells, as these non-structural proteins are crucial for the formation of ROs. Confocal results revealed that the JEV NS1 protein can effectively concentrate ER membranes (Fig. 3A through C). NS1', a 52-amino acid C-terminal extension of the NS1 protein, is an additional protein produced by the JE serogroup of orthoflavivirus through −1 programmed ribosomal frameshifting (–1PRF) (17, 18). Consequently, we investigated whether NS1' plays a similar role to NS1 in concentrating ER membranes. Confocal results indicated that the NS1' protein can also concentrate ER membranes (Fig. 3C and D), in contrast to the 52 additional amino acids produced by frameshifting, which do not exhibit this effect (Fig. 3D). Furthermore, the West Nile virus (WNV) NS1 protein was found to have a similar effect (Fig. 3E), suggesting that multiple orthoflavivirus NS1 proteins may concentrate ER membranes. Additionally, HeLa cells stably expressing RR-mNeonGreen (HeLa-RR) were generated using a lentiviral system (Fig. S2A). Consistent with the co-transfection results, both JEV infection and NS1 transfection were shown to concentrate ER membranes in HeLa-RR cells (Fig. S2B and C). Collectively, these findings suggest that the JEV NS1 protein plays a critical role in concentrating ER membranes to form MROs.

### The key sites of NS1 protein in ER membrane concentration

**Fig 4:**
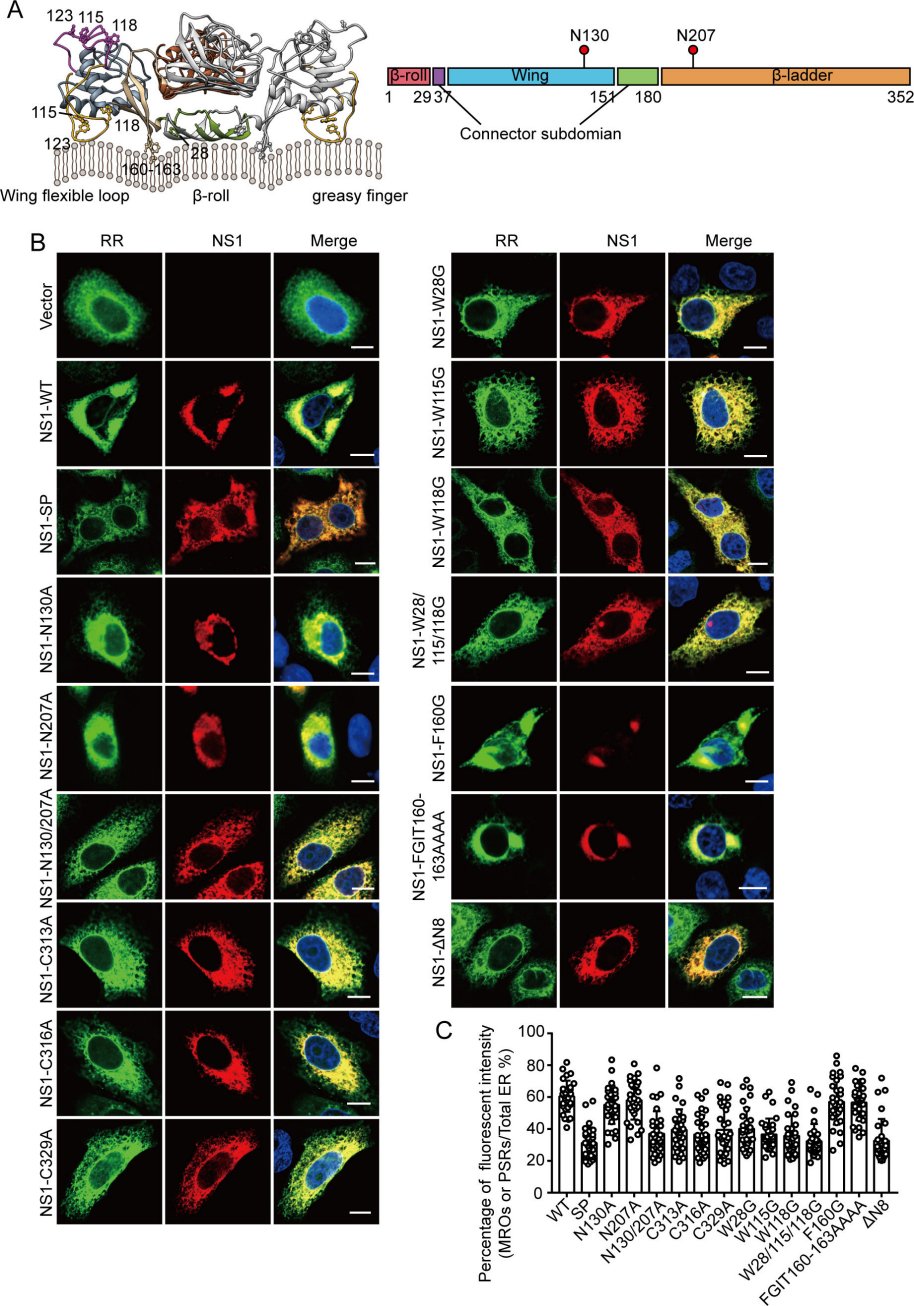
The key sites of NS1 protein in ER membrane concentration. (**A**) The structure of the JEV NS1. The structural model of NS1 dimer (left). The β-roll domain (amino acid residues 1–29) is colored in green, a wing domain (amino acids 38–151) is colored in blue, and β-ladder domains (amino acids 181–352) are colored in brown. Hydrophobic residues (namely, residues 28, 115, 118, 123, and 160–163) suspected to be involved with cell membrane interaction are labeled. This structural model was provided by Prof. Svetlana V. Antonyuk and adapted from reference 21. The primary structure of orthoflavivirus NS1 protein (right). The β-roll, wing, β-ladder domains, and two connector subdomains are marked in different colors. The glycosylation sites are shown with red dots. (**B**) HeLa cells were co-transfected with RR and mutant NS1 plasmids for 36 h. Immunofluorescence staining was performed to detect the ER membranes and viral NS1 protein. Scale bars, 10 µm. (**C**) Quantification of ER membrane concentration shown in B. Each point represents a single cell from two independent experiments. The statistics for WT NS1 are the same set as used in Fig. 3C.

During orthoflavivirus replication, the newly synthesized NS1 protein is translocated into the ER lumen via a signal peptide encoded by the final 24 amino acids of the E protein. The NS1 monomer undergoes modification with high-mannose carbohydrate moieties at the N130 and N207 sites and rapidly forms a dimeric species, acquiring hydrophobic characteristics (Fig. 4A) (19–21). Given the properties of the NS1 protein, we investigated the effects of NS1’s signal peptide, glycosylation sites (N130 and N207), oligomerization sites, and key hydrophobic sites on the ER membrane concentration. Initially, we replaced the last 24 amino acids of envelope protein (the translocating signal peptide of NS1) with the bovine preprolactin signal peptide sequence to effectively prevent the localization of NS1 protein in the ER lumen and designated this variant as NS1-SP. The absence of ER-localized signal peptides appears to have no impact on NS1 secretion; however, it significantly diminishes NS1’s ability to concentrate membranes (Fig. S3A, 4B and C). Subsequently, we introduced point mutations at the N130 and N207 sites of the NS1 plasmid (N130A, N107A, and N130/207A) to inhibit glycosylation. Following these mutations, the molecular weight of the mutant NS1 plasmids was slightly lower than that of WT plasmids, and the quantity of secreted NS1 (sNS1) in the supernatant correspondingly decreased (Fig. S3B). Single-point mutants at N130 or N207 were still capable of concentrating ER membranes, while the double mutation (N130/207A) impairs its ability to remodel the ER membranes (Fig. 4B and C). This impairment may be attributed to the double mutation significantly altering the solubility of the NS1 protein compared to the single mutation, resulting in reduced solubility within the ER lumen (22). Upon high-mannose carbohydrate modification, the NS1 monomer undergoes rapid dimerization, with the last three cysteines of NS1 (C313, C316, and C329) being essential for the oligomerization process (Fig. S3C) (19, 23). The C313A, C316A, and C329A mutations result in the inability of NS1 to form dimers and lead to the absence of sNS1 protein in the supernatant (Fig. S3D). The C313A, C316A, and C329A mutations significantly diminished the ability of NS1 to concentrate ER membranes (Fig. 4B and C), suggesting that dimers possess a greater capability for membrane remodeling compared to monomers. Finally, we investigated the impact of hydrophobic sites on NS1-concentrated ER membranes. Hydrophobic residues (W28, W115, W118, L123, and FGIT160-163) are suspected to be involved with membrane interaction (Fig. 4A; Fig. S3E) (21). The W28, W115, and W118 residues are highly conserved among various orthoflaviviruses (Fig. S3E). Mutations in these hydrophobic residues (W28G, W115G, W118G, and W28/115/118G) resulted in a marked loss of membrane-concentrating capacity (Fig. 4B and C), indicating that these three sites are essential for NS1 to concentrate membrane. However, the F160G and FGIT160-163AAAA mutants were able to concentrate ER membranes similarly to the WT NS1 protein (Fig. 4B and C). This finding parallels a study on DENV, where mutation at this loci (F160A) did not alter the lipid remodeling ability of NS1 (9). Interestingly, mutations in these membrane insertion sites did not affect NS1 secretion, suggesting that the NS1 secretion pathway may be independent of ER membrane insertion sites (Fig. S3A). The first eight amino acids at the N-terminus of the Zika virus (ZIKV) NS1 protein are believed to play a role in lipid binding (24). Similarly, the eight amino acids at the N-terminal of JEV NS1 are necessary for the concentration of the ER membrane. The absence of the eight amino acids (ΔN8) significantly impairs ER membrane remodeling (Fig. 4B and C), even though they are not conserved among orthoflaviviruses (Fig. S3E). The deletion of the N-terminal eight amino acids appears to preclude the protein from being secreted into the supernatants (Fig. S3A). These findings indicate that NS1 possesses complex functional characteristics, with certain regions contributing to protein secretion, others to the ER membrane concentration, and some involved in both pathways.

To further elucidate the key site of NS1 concentrating the ER membranes, the JEV NS1-3 and NS1-4A polyprotein plasmids were transfected into HeLa cells. The orthoflavivirus protease NS2B3 is responsible for cleaving viral precursor polyproteins (25). Thus, it was constructed in a plasmid designed to ensure that NS1 was cleaved into monomers, partially mimicking the production of the NS1 protein during viral infection. Confocal results demonstrated that the WT NS1-3 and NS1-4A polyprotein plasmids could concentrate ER membranes. In contrast, the mutant plasmids of NS1-3 and NS1-4A (N130/207A, W28/115/118G, and C313/316/329A) failed to induce the concentration of ER membranes (Fig. S4A and B). Collectively, our findings suggest that the ER-localized signal peptide of NS1, the high-mannose carbohydrate modification sites, the oligomerization sites, the hydrophobic amino acid sites, and the first eight amino acids at the β-roll are critical for NS1 to concentrate ER membranes. These sites and segments ensure that NS1 can accurately localize and form stable dimers for binding to ER membranes. In contrast, the FGIT160-163 sites may affect effective interactions with the transmembrane proteins of the ROs rather than altering the lipid-binding capacity of NS1 (9).

### NS1-induced ER membrane concentration **is independent of the cytoskeleton**

**Fig 5:**
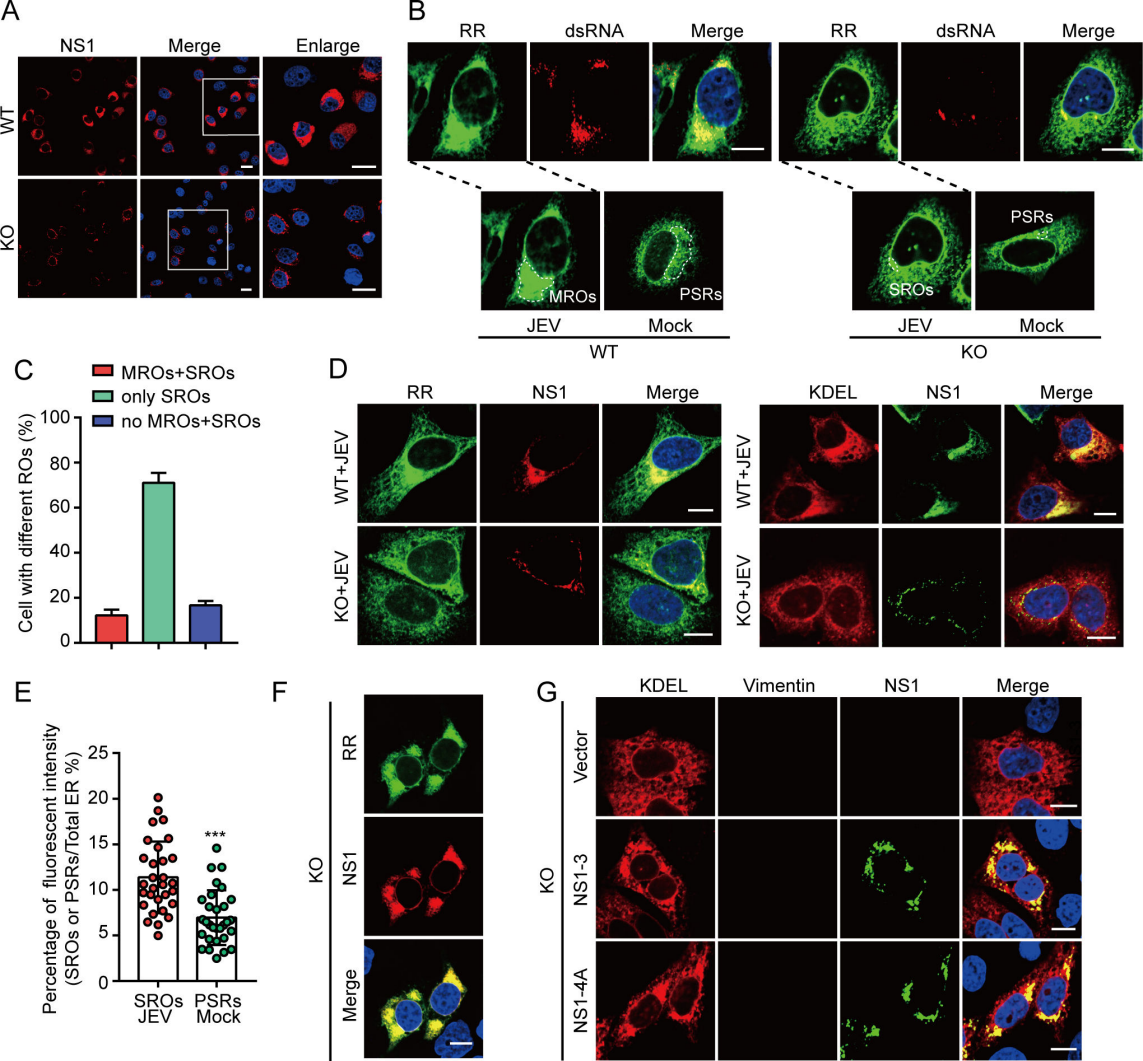
MROs are absent in Vimentin-deficient cells. (**A**) WT and Vim-KO HeLa cells were infected with the JEV NJ2008 strain, MOI = 0.5, 36 hpi. Immunofluorescence assays were performed to detect the JEV NS1 protein. Scale bars, 20 µm. (**B**) WT and Vim-KO HeLa cells were transfected with the RR plasmid and then infected with the JEV NJ2008 strain, MOI = 0.5, 36 hpi. Immunofluorescence assays were performed to observe the ER and viral dsRNA. Scale bars, 10 µm. MROs, SROs, and PSRs were indicated with dotted circles. (**C**) The percentage of JEV-infected Vim-KO HeLa cells with three different types of ER clusters. Average of three counts, each with more than 39 cells. (**D**) WT and Vim-KO HeLa cells were transfected with the RR or KDEL plasmid and then infected JEV NJ2008 strain, MOI = 0.5, 36 hpi. Immunofluorescence assays were performed to observe the ER and viral NS1 protein. Scale bars, 10 µm. (**E**) The percentage of ER fluorescence intensity in SROs or PSRs. Each point represents a single cell from two independent experiments, two-tailed *t*-test, ***P < 0.001. (**F**) Vim-KO HeLa cells were co-transfected with the RR and JEV NS1 plasmids. Immunofluorescence assays were performed to observe the ER and viral NS1 protein. Scale bars, 10 µm. (**G**) Vim-KO HeLa cells were co-transfected with KDEL and either the JEV NS1-3 or NS1-4A polyprotein plasmids. The concentration of ER membranes was observed using the confocal microscope. Scale bars, 10 µm.

The remodeling of ER membranes by viruses to form ROs is closely linked to cytoskeletal rearrangements (7, 26, 27). Our previous research has demonstrated that vimentin (Vim) can form a cage-like structure that envelops viral ROs to provide physical support for viral replication (28). Therefore, we focused on the effect of intermediate filament vimentin in the formation of MROs. In WT cells, a large number of viral proteins aggregate in the perinuclear region. In contrast, in vimentin knockout (Vim-KO) cells, only a minimal portion of viral protein is dispersed around the nucleus (Fig. 5A). This observation implies that the deletion of vimentin may obstruct the formation of MROs, resulting in the formation of only SROs. To investigate the formation of ER clusters (MROs or SROs) in JEV-infected vimentin-deficient cells, we examined the ER membranes using confocal microscopy on JEV-infected Vim-KO HeLa cells. Confocal imaging revealed that in WT cells, ER membranes aggregated to form MROs, which co-localized with dsRNA that clustered into sheets (Fig. 5B). Conversely, in Vim-KO cells, viral dsRNA appeared as dispersed spots around the nucleus, forming SROs instead of MROs (Fig. 5B and C). Notably, ER membranes were also concentrated in the SROs, although the area of infection was limited (Fig. 5D and E). These results suggest that the absence of vimentin inhibits the formation of MROs rather than impeding the remodeling of ER membranes induced by JEV. To test this conjecture, we transfected the NS1 protein into Vim-KO cells and examined the concentration of the ER membranes. Confocal results showed that NS1 can still concentrate ER membranes in Vim-KO cells (Fig. 5F and G). Additionally, withaferin A and acrylamide were used to disrupt the filament networks of vimentin (Fig. S5A and B) (29, 30). The treatments with withaferin A and acrylamide did not alter the concentration of ER membranes induced by NS1 (Fig. S5C and D). These results suggest that intermediate filament vimentin is not associated with NS1-mediated ER membrane concentration.

**Fig 6:**
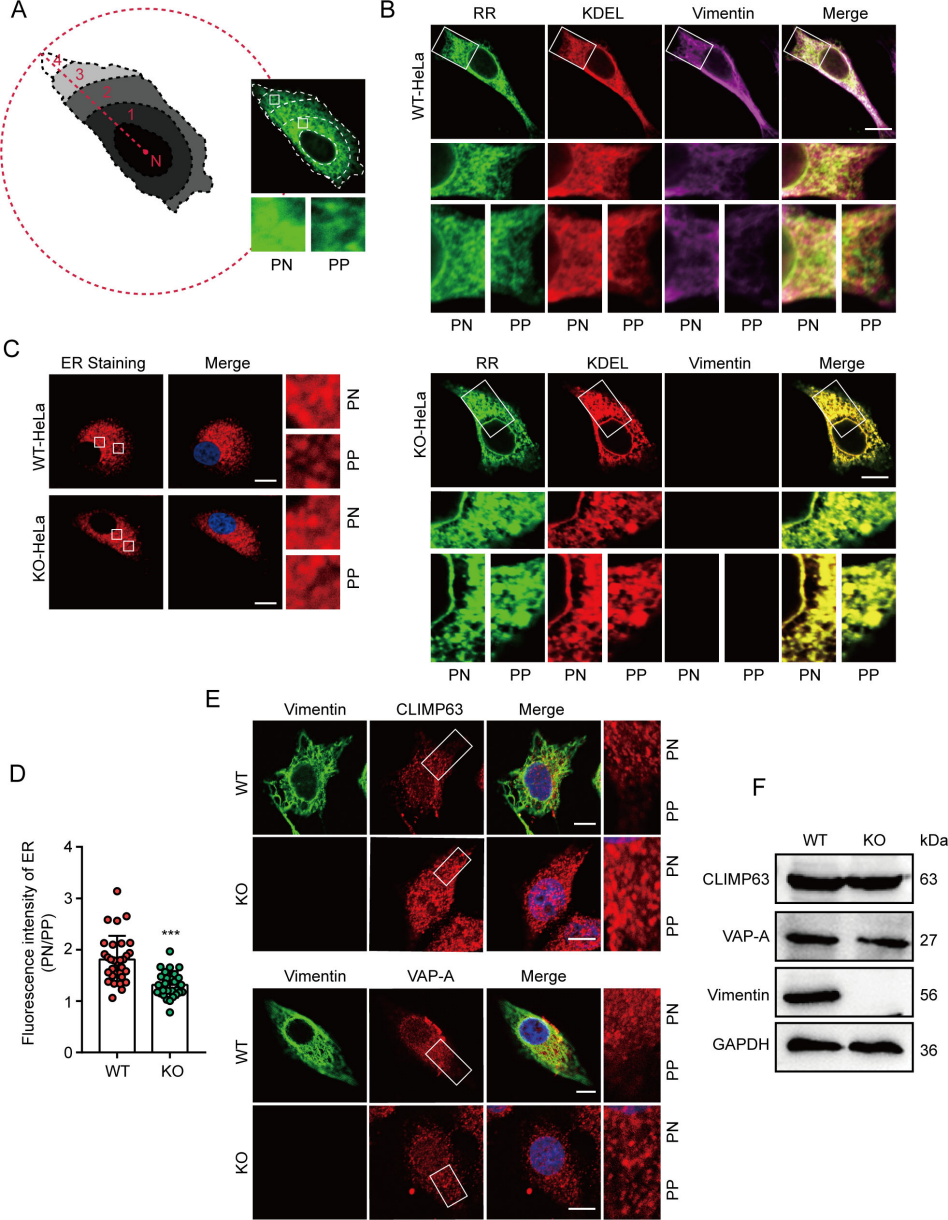
Vimentin regulates ER morphology. (**A**) The strategy for quantifying the ER membrane distribution (left). A demonstration of the distribution of the ER within a cell (right). PN, perinuclear; PP, cell periphery. (**B**) WT and Vim-KO HeLa cells were transfected with the RR and KDEL plasmids. Immunofluorescence assays were performed to observe the ER. Scale bars, 10 µm. (**C**) The ER of WT and Vim-KO HeLa cells stained with the dye was observed using confocal microscopy. Scale bars, 10 µm. (**D**) The quantification of the ER signal distribution in PN and PP regions. Each point represents a single cell from two independent experiments, two-tailed *t*-test, *** *P* < 0.001. (**E**) The immunofluorescence images of CLIMP63 and VAP-A in WT and Vim-KO HeLa cells. Scale bars, 10 µm. (**F**) The expression of CLIMP63 and VAP-A in WT and Vim-KO HeLa cells was detected by immunoblotting.

Since NS1-mediated ER membrane concentration is independent of vimentin, why are MROs not formed, but only SROs, in Vim-KO cells? We speculate that there are two reasons: (i) vimentin deficiency results in reduced viral replication, leading to insufficient NS1 protein to remodel ER membranes (28); (ii) in Vim-KO cells, the NS1 protein displayed a piecemeal distribution and cannot aggregate effectively to form MROs (Fig. 5A). Changes in the distribution of NS1 may be related to the subcellular localization of ER (31). Therefore, we explored whether vimentin affects the distribution of NS1 in JEV-infected cells by altering ER localization. We examined the morphological distribution of ER membranes in WT and Vim-KO cells (Fig. 6A). In WT cells, the ER extends throughout the cytoplasm, with membranes packing more densely near the PN compared to the highly dynamic tubule counterparts that reach into the cell periphery (PP; Fig. 6B through D). The concentration of ER membranes in the PN region of HeLa cells diminished in Vim-KO cells (Fig. 6B through D). Upon closer inspection of ER morphology, it became evident that ER membranes, which are normally retained in the PN region, had spread into the periphery of vimentin-KO cells (Fig. 6B). Additionally, we investigated the distribution of two ER membrane-associated endogenous proteins, CLIMP63 and VAP-A, in WT and Vim-KO HeLa cells. The deletion of vimentin leads to a marked redistribution of CLIMP63 and VAP-A toward the PP compared to WT cells, without affecting their overall abundance (Fig. 6E and F). These data suggest that the intimate connection between vimentin and the ER is essential for elaborate remodeling of the ER, which ensures the accumulation of the viral NS1 protein around the nucleus.

In addition to the intermediate filaments, we also examined the involvement of two other types of cytoskeletal components, microfilaments and microtubules, in the concentration of ER membranes induced by NS1. Treatment with nocodazole, a microtubule polymerization inhibitor, and latrunculin A, a microfilament-disrupting agent, did not impact the NS1-mediated concentration of the ER membranes (Fig. S5D through F). Interestingly, the four inhibitors altered the distribution of NS1 in JEV-infected cells as follows: (i) dimethyl sulfoxide (DMSO)-treated cells exhibited sheet-like infections and the formation of MROs in the perinuclear region; (ii) withaferin A- and acrylamide-treated cells displayed a punctate distribution of NS1 in the perinuclear region, consistent with JEV infection in Vim-KO cells; (iii) nocodazole and latrunculin A treatments resulted in NS1 diffusing throughout the cytoplasm, occasionally forming clumps resembling grape bunches. These variations in NS1 distribution suggest that three types of cytoskeleton may exert different effects on JEV replication. Despite the observed differences in distribution, ER membranes remain relatively concentrated in the region where NS1 is present (Fig. S5G). These results further indicate that the concentration of ER membranes induced by NS1 is independent of the cytoskeleton.

### MROs are efficient viral replication platforms

**Fig 7:**
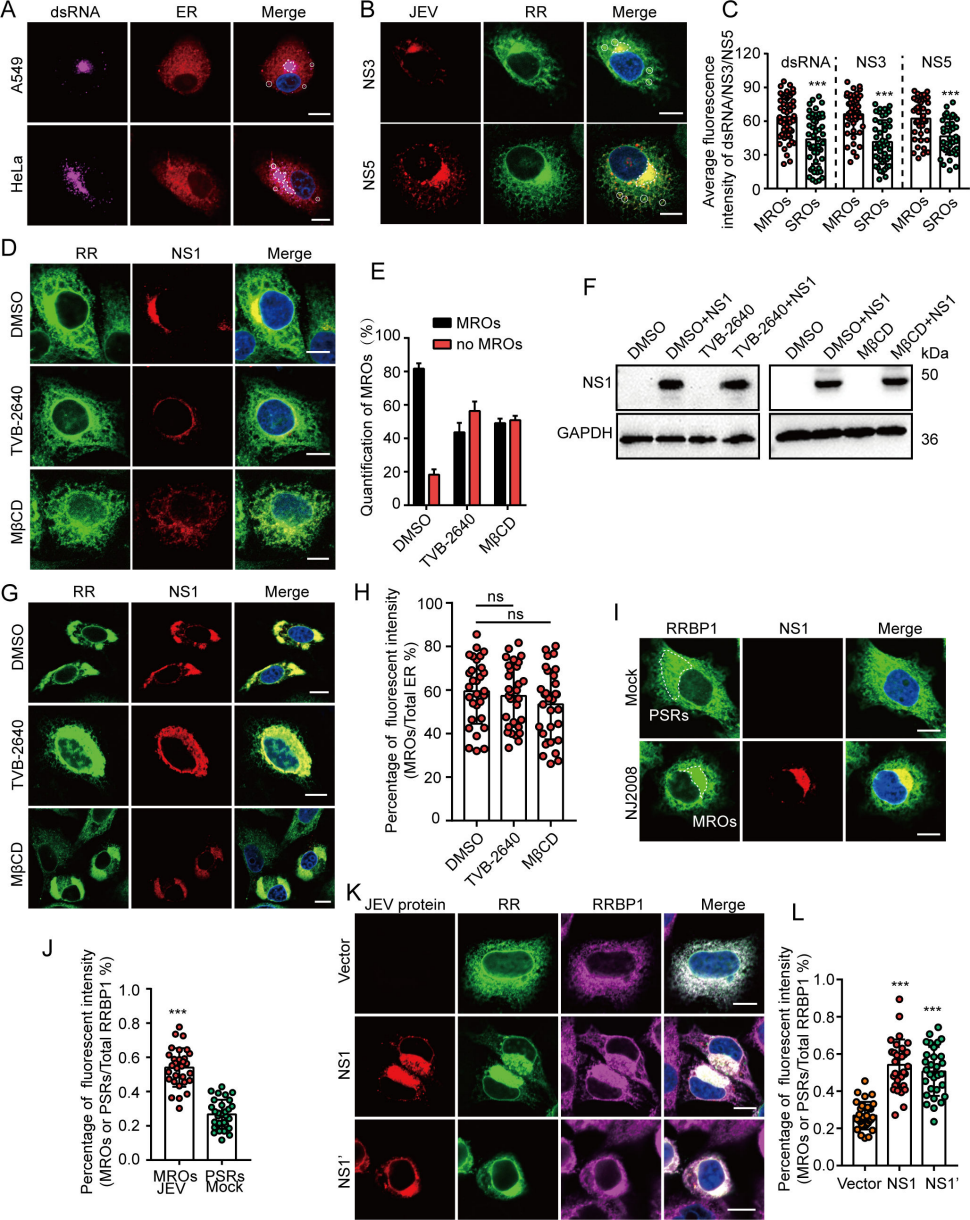
MROs enriched for host factors accelerate viral replication. (**A**) A549 and HeLa cells were infected with the JEV NJ2008 strain for 36 h at an MOI of 0.5. Viral dsRNA was stained with the corresponding antibody. The ER was stained with dye. The dotted circles represent MROs. Solid circles represent SROs. Scale bars, 10 µm. (**B**) HeLa-RR cells were infected with the JEV NJ2008 strain for 36 h at an MOI of 0.5. Viral NS3 and NS5 proteins were stained with the corresponding antibody. The dotted circles represent MROs. Solid circles represent SROs. Scale bars, 10 µm. (**C**) Analysis of dsRNA, NS3, and NS5 intensity per unit area in MROs and SROs, two-tailed *t*-test, ***P < 0.001. Each point represents a MRO or SRO from two independent experiments. (**D**) Immunofluorescence analysis of concentrated ER membranes induced by JEV infection in dimethyl sulfoxide (DMSO), TVB-2640 (1 µM), or MβCD (1 mM) treated HeLa-RR cells. MOI = 0.5, 36 hpi. Scale bars, 10 µm. (**E**) The percentage of JEV-infected cells with MROs following treatment with DMSO, TVB-2640 (1 µM), or MβCD (1 mM). Average of two counts, each with more than 39 cells. (**F**) The expression of NS1 in HeLa cells following treatment with TVB-2640 (1 µM) and MβCD (1 mM). (**G**) Immunofluorescence analysis of concentrated ER membranes induced by NS1 protein (plasmid transfection) in DMSO, TVB-2640 (1 µM), or MβCD (1 mM) treated HeLa-RR cells. Scale bars, 10 µm. (**H**) The percentage of ER fluorescence intensity in NS1-induced MROs after treatment of HeLa cells with DMSO, TVB-2640 (1 µM), or MβCD (1 mM). Each point represents a single cell from two independent experiments, two-tailed *t*-test, ns, not significant. (**I**) HeLa cells were infected with JEV NJ2008 strain, MOI = 0.5, 36 hpi. RRBP1 and viral NS1 protein were stained with respective antibodies. Scale bars, 10 µm. (**J**) The percentage of RRBP1 fluorescence intensity in MROs and PSRs. Each point represents a single cell from two independent experiments, two-tailed *t*-test, ***P < 0.001. (**K**) HeLa cells were co-transfected with the RR and either the NS1 or NS1' plasmids. RRBP1, NS1, and NS1' proteins were stained with respective antibodies. Scale bars, 10 µm. (**L**) The percentage of RRBP1 fluorescence intensity in MROs (NS1 or NS1' plasmids transfected cells) and PSRs (vector plasmid transfected cells). Each point represents a single cell from two independent experiments, two-tailed *t*-test, ***P < 0.001.

Next, we investigated whether the formation of MROs enhances viral replication. We assessed the average fluorescence intensity of dsRNA, NS3, and NS5 in both MROs and SROs. Our findings revealed that the intensity of dsRNA, NS3, and NS5 per unit area in MROs was stronger than that in SROs, indicating that viral replication was more efficient in MROs (Fig. 7A through C). Given that MROs are formed by the concentration of ER membranes, we further examined the roles of fatty acids and cholesterol, which are major components of ER membranes, in this process. The fatty acid synthase inhibitor TVB-2640 and cholesterol-depleting agent methyl-β-cyclodextrin (MβCD) significantly reduced viral replication (Fig. S6A through E). However, confocal microscopy revealed that in cells treated with TVB-2640 and MβCD, JEV infection can also lead to the concentration of ER membranes, although the formation of MROs was hindered due to the reduced level of viral infection (Fig. 7D and E). Additionally, TVB-2640 and MβCD treatment did not influence the expression of NS1 protein and the concentration of ER membranes induced by NS1 (Fig. 7F through H). These findings suggest that TVB-2640 and MβCD inhibited viral replication, resulting in a significant reduction in NS1 protein production and consequently impairing the formation of MROs, whereas TVB-2640 and MβCD did not affect NS1’s ability to concentrate ER membranes, i.e., the concentration of ER membranes induced by NS1 is not related to fatty acids and cholesterol.

**Fig 8:**
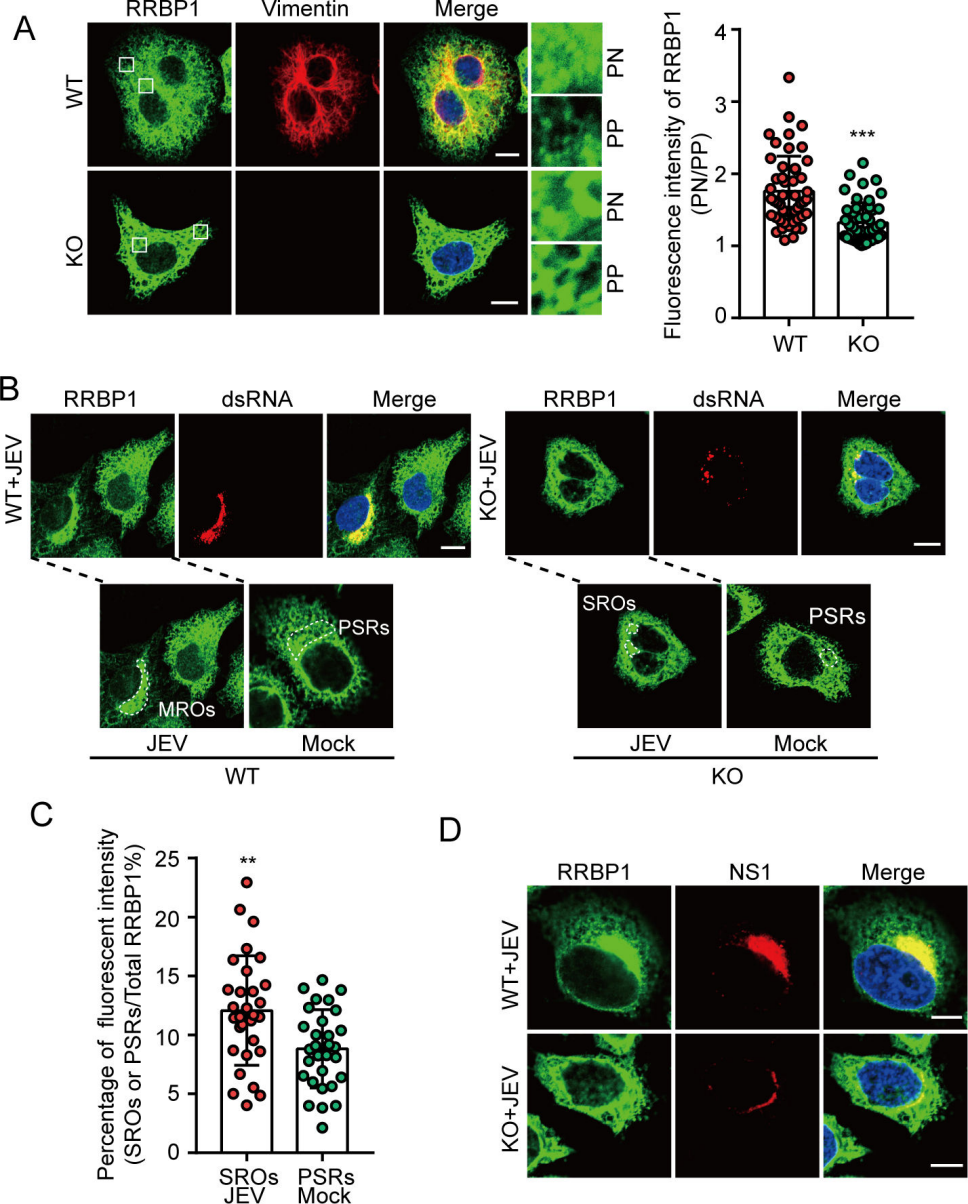
RRBP1 was concentrated in MROs and SROs. (**A**) The immunofluorescence images of RRBP1 in WT and Vim-KO HeLa cells (left). Scale bars, 10 µm. PN, perinuclear; PP, cell periphery. The quantification of RRBP1 signal distribution in PN and PP regions (right). Each point represents a single cell from two independent experiments, two-tailed *t*-test, ***P < 0.001. (**B**) WT and Vim-KO HeLa cells were infected with the JEV NJ2008 strain, MOI = 0.5, 36 hpi. Immunofluorescence assays were performed to observe RRBP1 and viral dsRNA. Scale bars, 10 µm. MROs, SROs, and PSRs were indicated with dotted circles. (**C**) The percentage of RRBP1 fluorescence intensity in SROs or PSRs. Each point represents a single cell from two independent experiments, two-tailed *t*-test, **P < 0.01. (**D**) WT and Vim-KO HeLa cells were infected with the JEV NJ2008 strain, MOI = 0.5, 36 hpi. Immunofluorescence assays were performed to observe RRBP1 and viral NS1 protein. Scale bars, 10 µm.

If MROs do provide a favorable environment for viral replication, it follows that host factors contributing to this process should be enriched within them. Thus, we explored whether MROs aggregate key host factors essential for viral replication. We focused on RRBP1, a ribosome-binding protein that interacts with viral dsRNA to promote viral replication (32). Consistent with previous findings, the knockdown of RRBP1 significantly suppressed JEV replication (Fig. S7A and B). In mock-infected cells, the distribution of RRBP1 resembled that of ER, whereas in JEV-infected cells, RRBP1 clustered in the perinuclear regions (Fig. 7I and J). NS1 and NS1' proteins also can aggregate RRBP1 in the perinuclear regions (Fig. 7K and L). Additionally, the distribution of RRBP1 was altered in a manner analogous to that of the ER membrane in Vim-KO cells (Fig. 8A). RRBP1 was also concentrated in SROs in JEV-infected Vim-KO cells (Fig. 8B through D). Collectively, these findings suggest that the MROs formed by JEV are enriched with host factors (e.g., RRBP1) essential for viral replication and promote rapid viral replication.

## DISCUSSION

Orthoflaviviruses are capable of forming ROs during ER replication. These virus-induced compartments enhance genome replication efficiency, facilitate the spatiotemporal coordination of the various stages of the viral replication cycle, and protect viral RNA from the hostile cytoplasmic environment (6, 7, 12). In this study, we demonstrate that NS1 effectively concentrates ER membranes in the perinuclear region to form MROs. This process relies on the ER-localized signal peptide, dimeric form, and the ER membrane-binding sites of the NS1 protein. The MROs are enriched with host factors essential for viral replication, such as RRBP1, and accelerate the replication process.

The biogenesis of orthoflavivirus ROs is predominantly influenced by the nonstructural proteins NS1, NS2A, NS2B, NS4A, and NS4B (7, 33). Our findings indicate that NS1 is a major protein that concentrates ER membranes (Fig. 3A through C). Viral replication relies on a continuous supply of ER membranes to provide energy and facilitate material exchange for ROs formation (34). Orthoflaviviruses concentrate ER membranes in the perinuclear region via NS1, which serves as a sufficient membrane source for the formation of ROs. ROs are subsequently formed through the induction of changes in ER membrane curvature, mediated by the nonstructural proteins NS2A, NS2B, NS4A, and NS4B (7, 33). Interestingly, it has been reported that other nonstructural proteins of orthoflaviviruses can remodel ER membranes, with NS4A being particularly notable for its ability to induce local membrane alterations (16, 35, 36). The expression of NS1 protein concentrates ER membranes in the perinuclear region to form MROs, whereas NS4A lacks this ability (Fig. 3A through C). This suggests that NS1 and NS4A differ in their mechanisms of ER membrane remodeling. It is very interesting to study the effects of different nonstructural proteins on the ER membrane. In addition, the interactions and synergies between different nonstructural proteins are crucial for the formation of the ROs. This highlights the need for a comprehensive study using a complex consisting of multiple factors.

Previous studies have demonstrated that the NS1 protein of ZIKV can remodel ER membranes (22, 24). Our study further identifies that the ER-localized signal peptide, glycosylation, dimeric form, and membrane binding sites are involved in this process. These sites are highly conserved across multiple orthoflaviviruses. Notably, the predicted membrane-binding site (residue 123) is not conserved among orthoflaviviruses (Fig. S3E). Structural studies of orthoflaviviruses have revealed that the flexible loop, along with the β-roll and connector subdomain, forms an extended hydrophobic protrusion, often referred to as a “spike,” which may be involved in membrane surface binding (9, 37, 38). Residue 123 is situated within the flexible loop, and its role in the concentration of ER membrane warrants further investigation. However, it is noteworthy that residues 122–124 consistently exhibit hydrophobic or positively charged in different orthoflaviviruses, regardless of amino acid variation, indicating that this region is likely involved in membrane binding and ER membrane concentration (Fig. S3E).

Our results also indicate that the concentration of ER membranes induced by NS1 is independent of the cytoskeleton (Fig. S5C, D and F). However, treatment with four cytoskeletal inhibitors demonstrated that NS1 exhibits a distinct morphological distribution and is unable to form MROs in cells infected with JEV (Fig. S5G). This phenomenon may be attributed to two factors: (i) the disruption of the cytoskeletal networks lead to a decrease in viral infection, which results in insufficient production of NS1 required for MROs formation (28, 39); (ii) the disruption of the cytoskeletal networks alters the morphology of the ER, thereby affecting the production and distribution of NS1 during viral infection (Fig. 6B through E) (40, 41). It is conceivable that vimentin organizes distinct ER substructural domains by selectively engaging with ER-associated binding partners (40). Vimentin deficiency disrupts the normal compartmental structure of the ER and prevents the aggregation of the NS1.

A limitation of this study is that we did not use live virus mutations to investigate the impact of key NS1 sites on RO formation and viral replication. Instead, we relied only on mutant polyprotein plasmids to partially simulate the viral replication process. Future studies should employ infectious clones or replicons to confirm the effects of the NS1 locus on the centralization of ER membranes.

In conclusion, our findings indicate that orthoflaviviruses utilize NS1 to concentrate the ER membranes, thereby ensuring an adequate membrane source for efficient viral replication (Fig. S8A through C).

## MATERIALS AND METHODS

### Cells, viruses, and plasmids

HeLa, 293T, A549, Vero, Neuro-2a, BHK-21, PK-15, DF-1, and C6/36 cells were cultured in Dulbecco’s modified Eagle’s medium (DMEM, 11965092, Gibco). Vimentin KO HeLa cells were kindly provided by Prof. Xiuli Feng (Nanjing Agricultural University, Nanjing, China) (42). Media were supplemented with 10% fetal bovine serum (FBS, 10100147C, GIBCO) and 1% penicillin-streptomycin. All cells, except C6/36, were cultured at 37°C with 5% CO_2_. C6/36 cells were cultured at 28°C with 5% CO_2_. JEV NJ2008 (GenBank version no. GQ918133.2), SA14-14-2 (GenBank version no. MK585066.1), HEN0701 (GenBank version no. FJ495189.1), and TMUV XZ2012 (GenBank version no. KM188953.1) were preserved in our laboratory. All JEV strains used in this manuscript were propagated in C6/36 cells. TMUV used in this manuscript was propagated in BHK-21 cells. The titers of the virus were titrated with plaque assays.

The mCherry-KDEL and RR-mNeonGreen plasmids were kindly provided by Prof. Lei Shi (Institute of Basic Medical Sciences, Chinese Academy of Medical Sciences and School of Basic Medicine, Beijing, China) (13, 15). The JEV NS1-SP plasmid was constructed based on a pcDNA3.1-ss-Myc ZIKV NS1 plasmid (gifted by Prof. Lei Shi, Institute of Basic Medical Sciences, Chinese Academy of Medical Sciences and School of Basic Medicine, Beijing, China), which was carrying an N-terminal bovine preprolactin signal peptide sequence and Myc sequence (24).

The plasmid construction of JEV proteins (NS1, NS1’, NS2B, NS3, and NS5) was performed as previously described (43). Genes of JEV NS1-3 or NS1-4A were inserted into the pcDNA3.1+ to construct the NS1-3 and NS1-4A polyprotein plasmids. NS1, NS1-3, and NS1-4A mutant plasmids were constructed based on the wild-type plasmid using a homologous recombination method. The JEV NS2A-HA plasmid was kindly provided by Dr. Jianchao Wei (Shanghai Veterinary Research Institute, Shanghai, China) (44). The JEV NS4A-eGFP, NS4B-mCherry, and WNV-NS1-HA plasmids were kindly provided by Prof. Bo Zhang (Wuhan Institute of Virology, Wuhan, China) (45, 46).

### Antibodies and reagents

As previously described, mouse monoclonal antibodies against JEV NS1, NS1', and TMUV prM proteins were generated in our laboratory (17). Commercial reagents used in this study are as follows: dsRNA (J2) mouse monoclonal antibody (10010200, Nordic MUbio), JEV NS3 protein rabbit polyclonal antibody (GTX125868, GeneTex), JEV NS4B protein rabbit polyclonal antibody (GTX125865, GeneTex), JEV NS5 protein rabbit polyclonal antibody (GTX131359, GeneTex), RRBP1 rabbit polyclonal antibody (22015–1-AP, Proteintech), vimentin rabbit monoclonal antibody (ab92547, Abcam), vimentin mouse monoclonal antibody (BF8006, Affinity), CLIMP63 mouse monoclonal antibody (sc-393544, Santa cruz), VAP-A mouse monoclonal antibody (sc-293278, Santa cruz), HA tag rabbit polyclonal antibody (51064–2-AP, Proteintech), FLAG (DYKDDDDK) tag mouse monoclonal antibody (66008–4-Ig, Proteintech), GAPDH mouse monoclonal antibody (AC033, ABclonal), goat anti-rat IgG Alexa Fluor 555 (A-21434, Invitrogen), goat anti-rabbit IgG Alexa Fluor 488 (A-11008, Invitrogen), donkey anti-rabbit IgG Alexa Fluor 647 (ab150075, Abcam), goat anti-mouse IgG Alexa Fluor 647 (ab150115, Abcam), goat anti-rabbit IgG Alexa Fluor 594 (ab150080, Abcam), goat anti-mouse IgG Alexa Fluor 488 (ab150113, Abcam), goat anti-rabbit IgG-horseradish peroxidase (HRP; 31460, Invitrogen), goat anti-mouse IgG-HRP (31430, Invitrogen), ER Staining kit-Red Fluorescence-Cytopainter (ab139482, Abcam), DMSO (M3850, AbMole), acrylamide (M11469, AbMole), withaferin A (M7490, AbMole), latrunculin A (ab144290, Abcam), nocodazole (M3194, AbMole), MβCD (M11339, AbMole), and TVB-2640 (M10746, AbMole).

### RNA interference, plasmid transfection, and JEV infection

For RNA interference, cells were seeded in 24-well plates overnight and transfected with the indicated small interfering RNA (siRNA) using the Lipofectamine 3000 transfection kit (L3000015, Invitrogen) for 48 h. The siRNA primer sequence of RRBP1 used in this study is as follows: 5'GAUGAGAUUCAGAAUAUGATT3'. For plasmid transfection, cells were transfected with indicated plasmids using the Lipofectamine 3000 transfection kit according to the instructions. For JEV infection assays, cells were inoculated with JEV at the indicated MOI in FBS-free medium at 37°C. After 2 h, the medium was supplanted with fresh DMEM containing 2% FBS, and incubation was continued for the indicated times.

### Western blotting

For denaturing SDS-PAGE gel, cells were lysed in RIPA buffer (89900, Thermo Fisher) at 4°C for 20 min. The protein samples were boiled for 5 min and added to the SDS loading buffer. For the western blotting of NS1 dimers, cells were lysed in nondenaturing lysis buffer (SL1030, Coolaber) at 4°C for 15 min. The protein samples (without heating) were added to a nondenaturing loading buffer (SL11704, Coolaber). The Equal amounts of proteins were subjected to SDS-PAGE and transferred to the polyvinylidene difluoride membrane. The membranes were incubated with indicated primary antibodies overnight at 4°C after being blocked with 5% nonfat milk for 2 h at room temperature (RT). For chemiluminescent readout, the membranes were incubated with HRP-conjugated IgG secondary antibodies for 1 h at RT and exposed using BIO-RAD Clarity Western ECL substrate.

### Confocal microscopy assays

Cells were fixed with 4% paraformaldehyde for 20 min at RT and permeabilized with 0.1% Triton X-100 for 10 min at RT. Subsequently, cells were blocked with 1% bovine serum albumin (BSA) for 1 h. Next, cells were incubated with indicated primary antibodies at 4°C overnight, followed by incubation with secondary antibodies in the dark at 37°C for 1 h. For ER staining, cells were stained with ER dye in the dark at 37°C for 40 min. The nucleus was stained with 4,6-diamidino-2-phenylindole at RT for 5 min.

### Transmission electron microscopy

Briefly, cells were infected with JEV for 36 h and then fixed with 2.5% glutaraldehyde in cacodylate buffer for 60 min at RT. The samples were sent to the Shanghai Veterinary Research Institute (Shanghai, China) for subsequent section preparation and electron microscopic analysis.

### Quantitative reverse transcription PCR

Cells were lysed by TRIzol (9109, TaKaRa), and the extracted RNA was introduced for reverse transcription using HiScript III RT SuperMix (R323-01, Vazyme) according to manufacturer protocol. Quantitative reverse transcription PCR (qRT-PCR) was performed with an SYBR green qPCR kit (711–02, Vazyme) on a QuantStudio 6 real-time PCR system. The 2^−ΔΔCt^ method was adopted to analyze the relative gene expression, and the qRT-PCR data were collected from three independent experiments. The primer sequences for this study are as follows:

NJ2008-E-F 5'GGCAAACGACAAACCAACATT3'

NJ2008-E-R 5'ATCAGCTCGCTTCTCGTTGTG3'

GAPDH-F 5'GAGTCAACGGATTTGGTCGT3'

GAPDH-R 5'GACAAGCTTCCCGTTCTCAG3'.

### Cell viability assays

Cells were seeded in 96-well plates and then treated with inhibitors for 24 h. The cytotoxic effect of the inhibitors was evaluated using a Cell Counting Kit-8 (CCK8; A311-01, Vazyme).

### Stable cell line

The RR-mNeonGreen gene was subcloned into the pCDH vector (pCDH-RR). 293T cells were transiently co-transfected with psPAX2, pMD2.G, and pCDH-RR plasmids. After 8 h of transfection, the medium was replaced with a fresh culture medium. After culturing for 72 h, the growth medium containing lentivirus was collected. HeLa cells were infected with lentiviruses in the presence of polybrene (10 µg/mL). After 48 h of infection, cells were selected by 10% FBS-DMEM containing puromycin (3 µg/mL) for 2 weeks to acquire stable cell lines.

### Categorization of ER clusters and viral component quantification

The categorization of ER clusters is as previously described (11). Briefly, the ER channel was filtered using Gaussian Blur with a Sigma (Radius) of 5.00 (MROs) or 2.00 (SROs) in ImageJ. A binary image was then created by manually setting the threshold and selecting the regions manually. The regions with sizes ranging from 50 to 300 µm^2^ were classified as MROs, while those smaller than 50 µm^2^ were categorized as SROs. The fluorescence intensity of dsRNA, NS3, and NS5 in both MROs and SROs was measured by dividing the integrated intensity within each MRO or SRO by the respective area.

### Quantification of ER clusters

In JEV-infected cells, MROs and SROs were manually circled using ImageJ. The percentage of the MROs area was calculated by dividing the ER cluster area by the total ER area. The percentage of MROs and SROs fluorescence intensity was calculated by dividing the ER cluster fluorescence intensity by the total ER fluorescence intensity. For mock cells, we manually circled a region in the perinuclear area with the same proportion of MROs or SROs as in JEV-infected cells, which we designated as the PSRs. The fluorescence intensity of these regions as a percentage of the whole ER was calculated by ImageJ.

In plasmid-transfected cells, if there are MROs present, they were manually circled, and the corresponding area and fluorescence intensity were calculated. If there is no MRO, PSRs were circled manually based on the percentage of MRO area in positive cells (cells expressing NS1). The ER concentrations induced by individual JEV nonstructural proteins or NS1 mutants were quantified by calculating the fluorescence intensity of MROs or PSRs as a percentage of total ER.

### ER membrane distribution analysis

The distribution analysis of ER membranes is as previously described (40). Briefly, in the ImageJ plugin, we segmented cell nuclei using the thresholding method followed by optional manual correction. The outer edges of the cells were manually labeled. We then used Euclidean distance maps to measure the relative distance from each pixel within the cytoplasm (outside and inside the nucleus) to the nucleus (normalized to the maximum distance per cell). These distance values were then partitioned into optional four partitions. The total signal of each partition was divided by the number of pixels to give the fluorescence intensity. The fluorescence intensity value was compared across bins 1 and 3.

### Amino acid sequence comparison

The NS1 amino acid sequences of the JEV and other representative orthoflavivirus were compared using the MegAlign program in DNAStar. The four dengue serotypes: dengue virus type 1 (DENV-1, MW288036.1), dengue virus type 2 (DENV-2, KM204118.1), dengue virus type 3 (DENV-3, MW288040.1), and dengue virus type 4 (DENV-4, KJ596664.1). The Spondwenii serocomplex: ZIKV (MN577550.1) and Spondwenii virus (NC_029055.1). A distinct serotype: Yellow fever virus (AY640589.1). The JE serocomplex: WNV (HQ596519.1), Murray Valley encephalitis virus (NC_000943.1), Usutu virus (NC_006551.1), Kunjin virus (KT934804.1), Alfuy virus (Alfuy, AY898809.1), and JEV (GQ918133.2).

### Statistical analysis

Statistical analysis was performed by an unpaired two-tailed *t*-test and expressed as the mean ± SD. A *P* value of <0.05 was considered statistically significant.

## Data Availability

All data supporting the findings of this study are included within the article and its supplemental material. The original data can be provided upon request to the corresponding author, Ruibing Cao (crb@njau.edu.cn).

## SUPPLEMENTAL MATERIAL

Figure S1 jvi.02113-24-s0001.tif Labeling ER with RR-mNeonGreen.

Figure S2 jvi.02113-24-s0002.tif JEV concentrates ER in the stable cell line expressing RR.

Figure S3 jvi.02113-24-s0003.tif Secretion of NS1 mutants.

Figure S4 jvi.02113-24-s0004.tif Polyprotein plasmids mimic JEV nonstructural protein production.

Figure S5 jvi.02113-24-s0005.tif NS1-induced ER membrane concentration is independent of the cytoskeleton.

Figure S6 jvi.02113-24-s0006.tif TVB-2640 and MβCD inhibit JEV replication.

Figure S7 jvi.02113-24-s0007.tif Knockdown of RRBP1 inhibits JEV replication.

Figure S8 jvi.02113-24-s0008.tif Model of the ER membrane concentration induced by NS1.

Supplemental legends jvi.02113-24-s0009.docx Legends for Fig. S1 to S8.

ASM does not own the copyrights to Supplemental Material that may be linked to, or accessed through, an article. The authors have granted ASM a non-exclusive, world-wide license to publish the Supplemental Material files. Please contact the corresponding author directly for reuse.
