# Supplementary figures and images for: The Japanese encephalitis virus NS1 protein concentrates ER membranes in a cytoskeleton-independent manner to facilitate viral replication
Source: J Virol. 2025 Feb 5;99(3):e02113-24. doi: 10.1128/jvi.02113-24 (PMC11915877; doi:10.1128/jvi.02113-24)

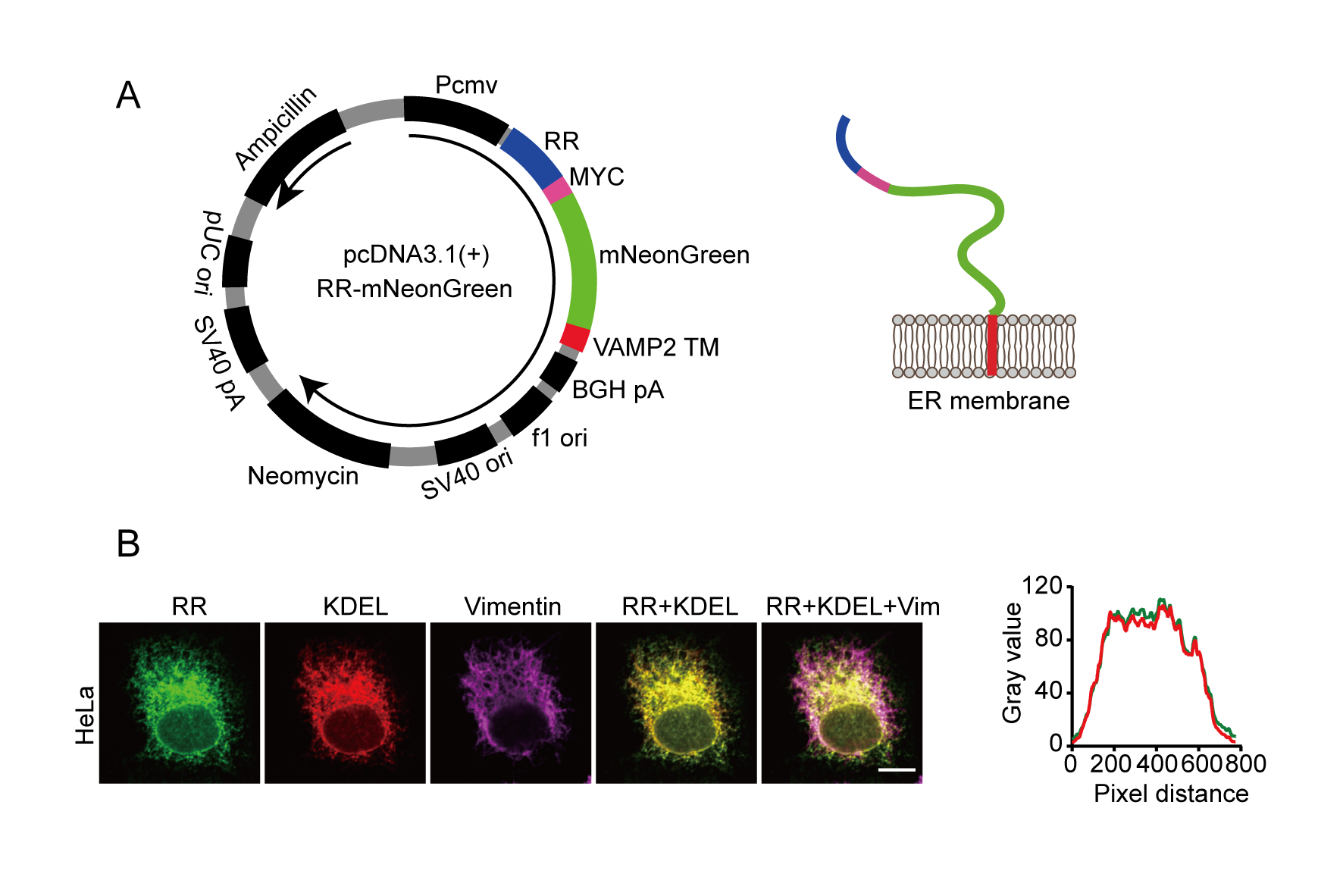

Supplement: Figure S1 — Labeling ER with RR-mNeonGreen. [file jvi.02113-24-s0001.tif]

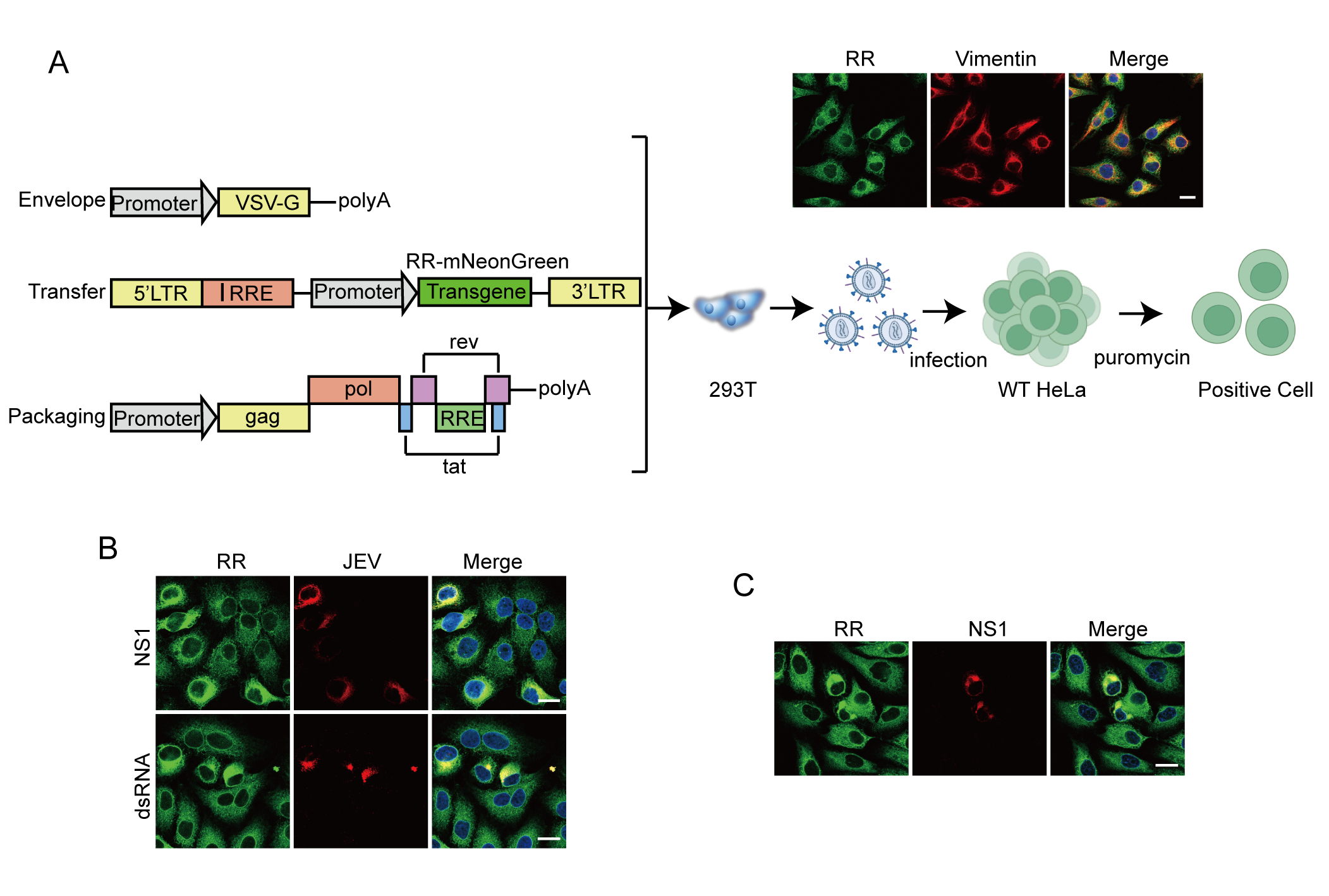

Supplement: Figure S2 — JEV concentrates ER in the stable cell line expressing RR. [file jvi.02113-24-s0002.tif]

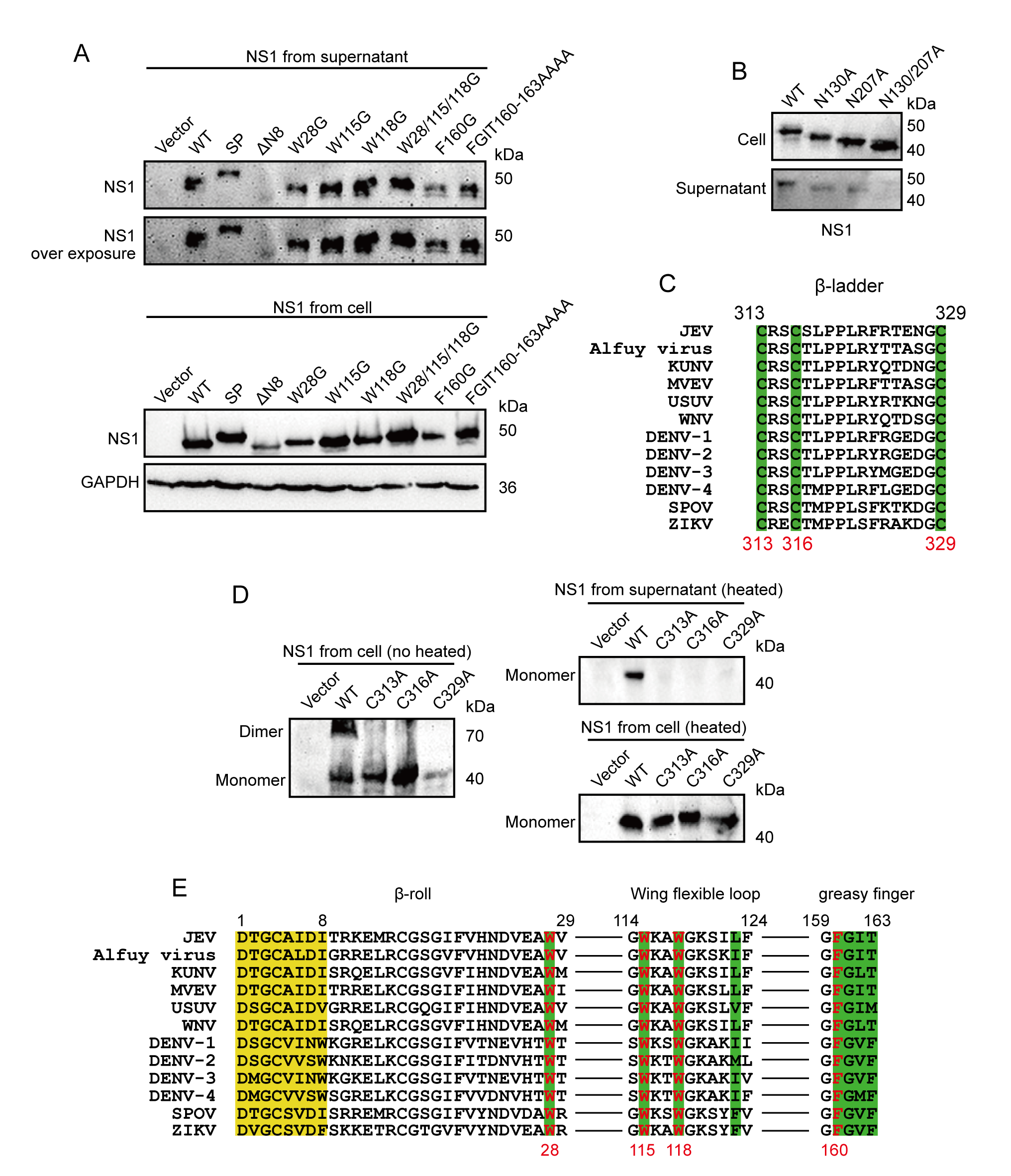

Supplement: Figure S3 — Secretion of NS1 mutants. [file jvi.02113-24-s0003.tif]

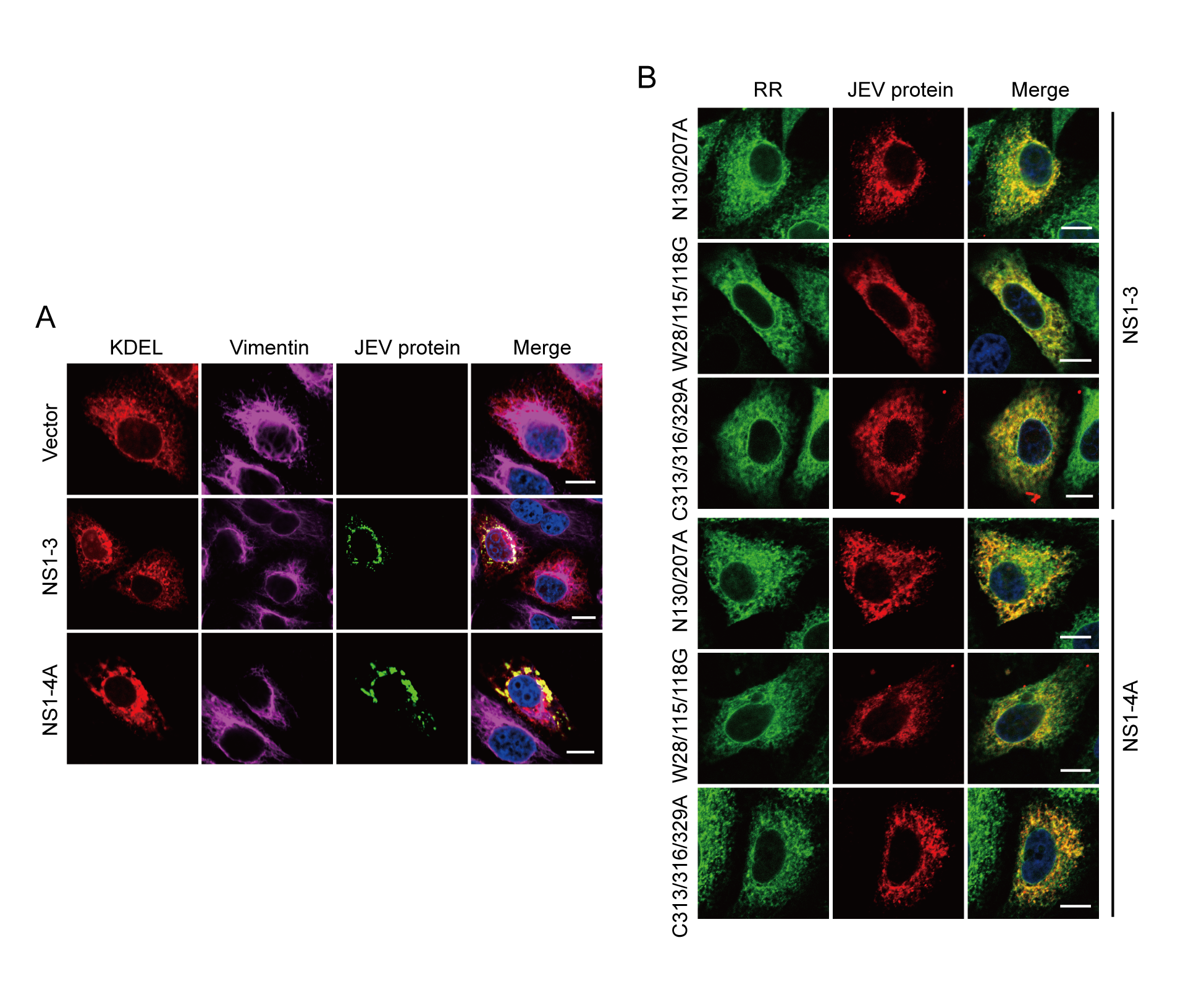

Supplement: Figure S4 — Polyprotein plasmids mimic JEV nonstructural protein production. [file jvi.02113-24-s0004.tif]

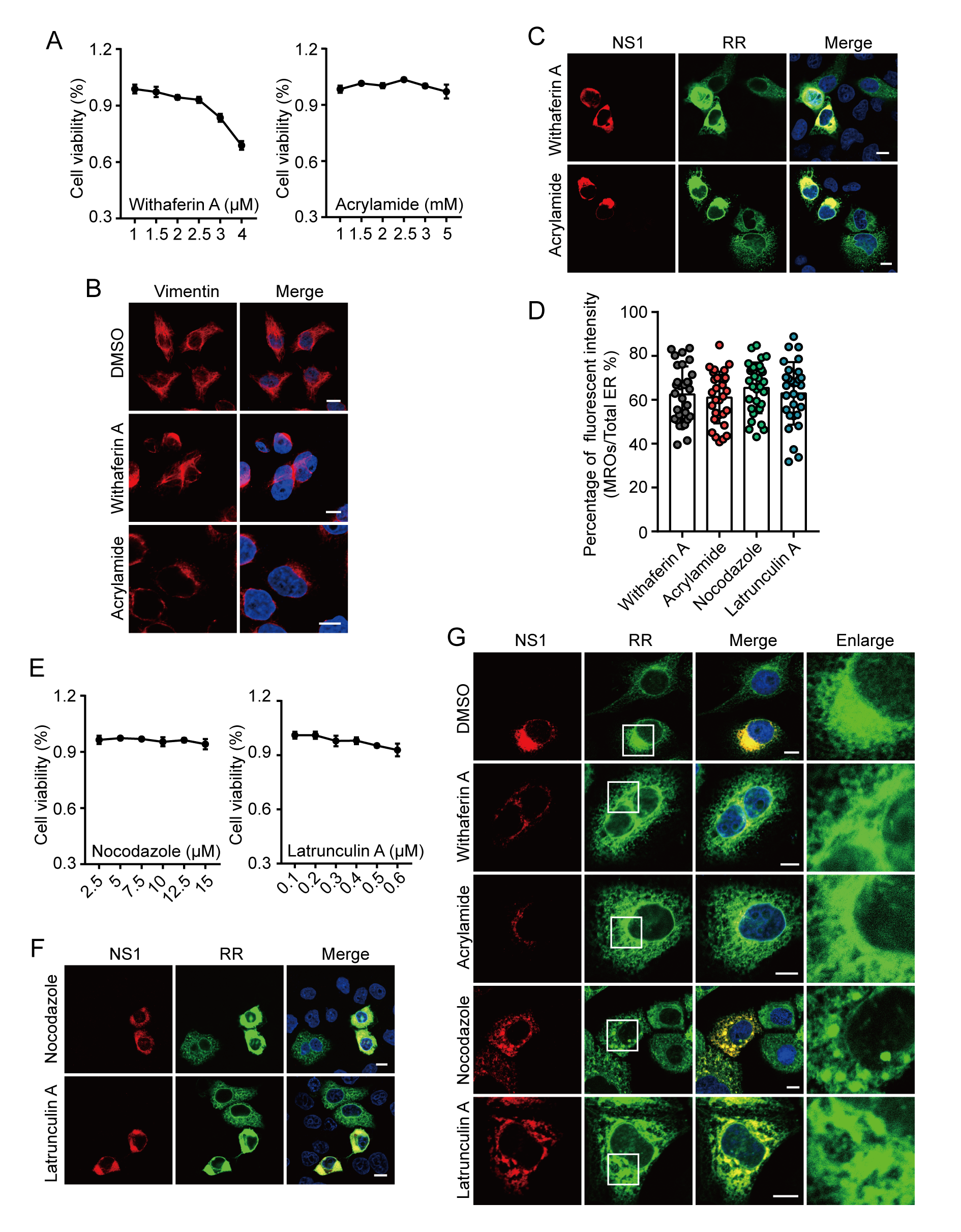

Supplement: Figure S5 — NS1-induced ER membrane concentration is independent of the cytoskeleton. [file jvi.02113-24-s0005.tif]

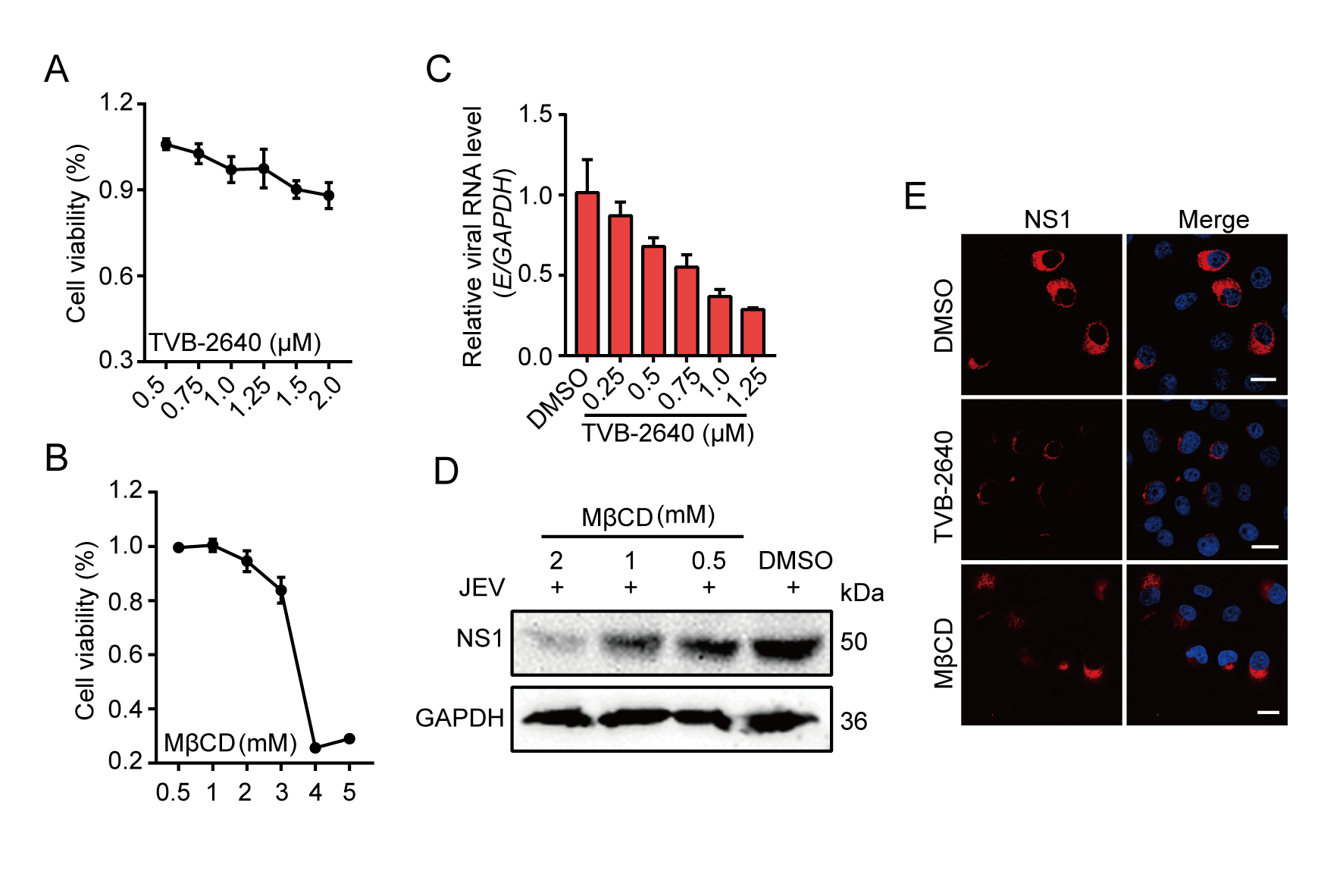

Supplement: Figure S6 — TVB-2640 and MβCD inhibit JEV replication. [file jvi.02113-24-s0006.tif]

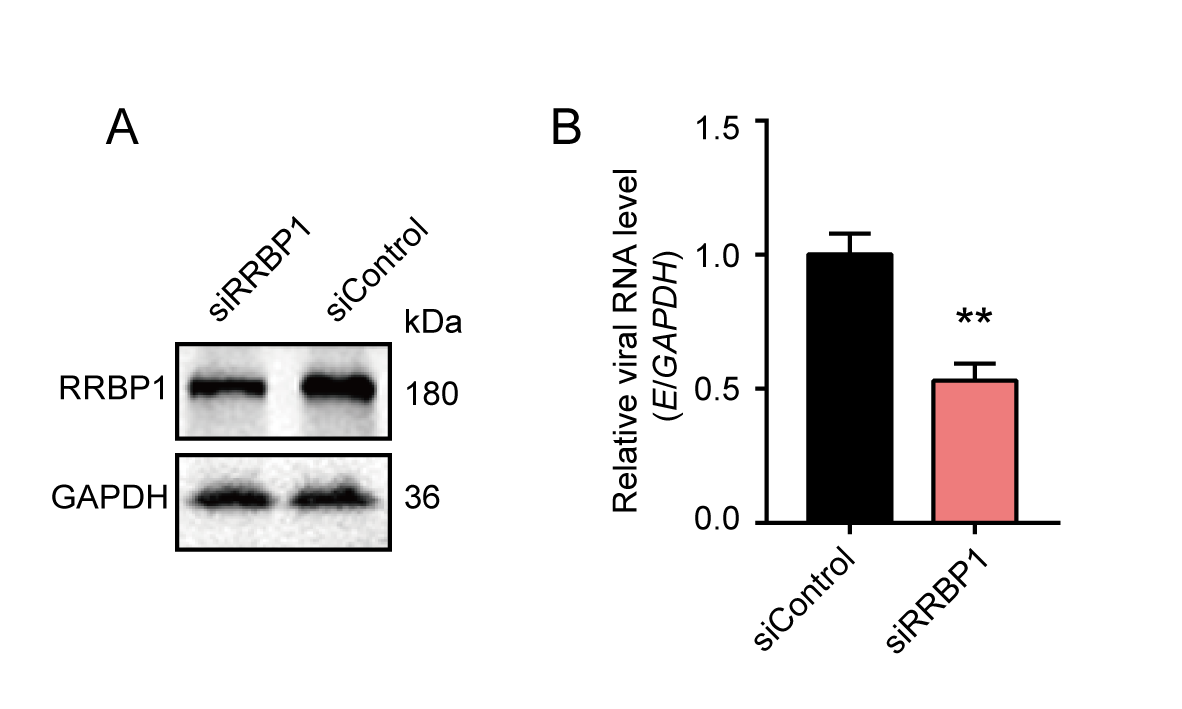

Supplement: Figure S7 — Knockdown of RRBP1 inhibits JEV replication. [file jvi.02113-24-s0007.tif]

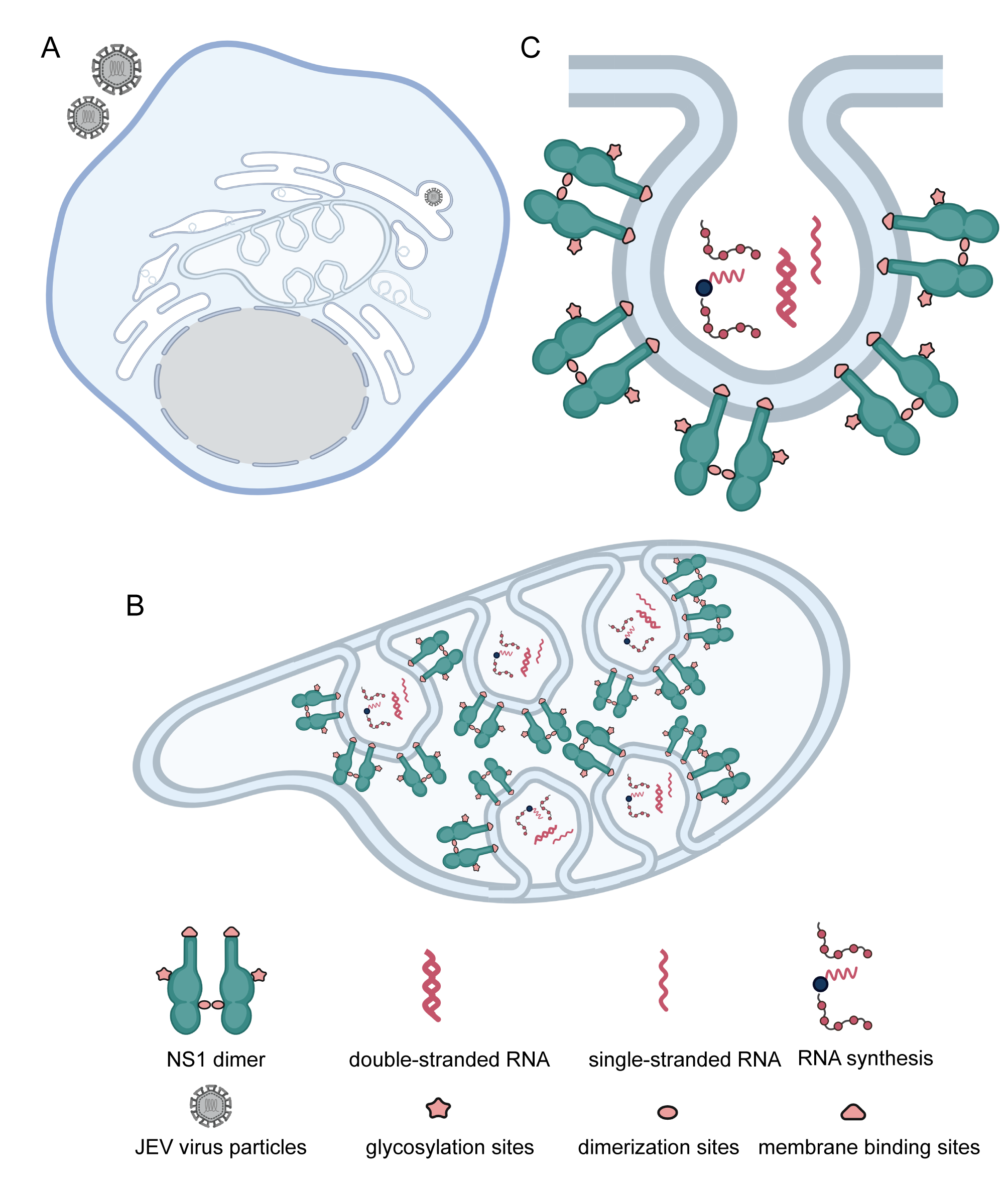

Supplement: Figure S8 — Model of the ER membrane concentration induced by NS1. [file jvi.02113-24-s0008.tif]
