# Supplementary material for: The Japanese encephalitis virus NS1 protein concentrates ER membranes in a cytoskeleton-independent manner to facilitate viral replication
Source: J Virol. 2025 Feb 5;99(3):e02113-24. doi: 10.1128/jvi.02113-24 (PMC11915877; doi:10.1128/jvi.02113-24)
Supplement: Supplemental legends — Legends for Fig. S1 to S8. [file jvi.02113-24-s0009.docx]

**Supplemental information**

**Figure S1. Labeling ER with RR-mNeonGreen**

(**A**) The map of plasmid encoding RR-mNeonGreen (left). The model diagram of RR-mNeonGreen labeled ER (right). (**B**) The immunofluorescence images of RR and KDEL in HeLa cells (left). Scale bars, 10 µm. Vim, vimentin. The co-localization analysis of RR and KDEL (right).

**Figure S2. JEV concentrates ER membranes in the stable cell line expressing RR**

(**A**) HeLa cells stably expressing RR (HeLa-RR) were constructed using a lentiviral system. (**B**) HeLa-RR cells were infected with the JEV NJ2008 strain at an MOI of 0.5 for 36 h. The concentration of ER membranes was observed using the confocal microscope. Scale bars, 20 µm. (**C**) HeLa-RR cells were transfected with the JEV NS1 plasmid for 24 h. The concentration of ER membranes was observed using the confocal microscope. Scale bars, 20 µm.

**Figure S3. The secretion of NS1 mutants**

(**A** and **B**) The expression (cell) and secretion (supernatant) of WT and mutant NS1 protein were detected by western blotting. Signal peptide deletion (SP), N-terminal eight amino acid deletion (ΔN8), W28G, W115G, W118G, W28/115/118G, F160G, and FGIT160-160AAAA mutants were shown in A. N130A, N207A, and N130/207A mutants were shown in B. (**C**) The comparison of residues 313-329 in different orthoflavivirus NS1 proteins. 313, 316, and 329 residues in these regions are highly conserved. (**D**) Western blotting analysis of NS1 dimers and monomers in cells transfected with WT and mutant NS1 plasmids (left). Western blotting analysis of the expression (cell) and secretion (supernatant) of WT and mutant NS1 protein (right). (**E**) Alignment of hydrophobic regions among orthoflavivirus NS1 sequences. Multiple hydrophobic residues in these regions are highly conserved.

**Figure S4. Polyprotein plasmids mimic JEV nonstructural protein production**

(**A**) HeLa cells were co-transfected with polyprotein plasmids encoding either NS1-3 or NS1-4A, along with KDEL plasmids. The concentration of ER membranes was observed using the confocal microscope. Scale bars, 10 µm. (**B**) HeLa-RR cells were transfected with mutant NS1-3 and NS1-4A plasmids. The concentration of ER membranes was observed using the confocal microscope. Scale bars, 10 µm.

**Figure S5. NS1-induced ER membrane concentration is independent of the cytoskeleton**

(**A**) Cell viability was measured using CCK8 assays in HeLa cells treated with withaferin A and acrylamide. (**B**) HeLa cells were treated with withaferin A (2 μM) and acrylamide (2.5 mM) for 24 h. Vimentin in cells was analyzed using the confocal microscope. Scale bars, 10 µm. (**C**) HeLa cells were cotransfected with the RR and JEV NS1 plasmids and then treated with withaferin A (2 μM) and acrylamide (2.5 mM) for 24 h. The NS1-mediated ER membrane concentration was observed using confocal microscopy. Scale bars, 10 µm. (**D**) The quantification of NS1-induced ER membrane concentration following treatment of HeLa cells with the indicated inhibitors. Each point represents a single cell from two independent experiments. (**E**) Cell viability was measured using CCK8 assays in HeLa cells treated with nocodazole and latrunculin A. (**F**) HeLa cells were cotransfected with the RR and JEV NS1 plasmids and then treated with nocodazole (10 μM) or latrunculin A (0.4 μM) for 24 h. The NS1-mediated ER membrane concentration was observed using confocal microscopy. Scale bars, 10 µm. (**G**) JEV-infected HeLa-RR cells (MOI = 1) were treated with withaferin A (2 μM), acrylamide (2.5 mM), nocodazole (10 μM), or latrunculin A (0.4 μM) for 24 h. The NS1 distribution and ER membrane concentration were observed using the confocal microscope. Scale bars, 10 µm.

**Figure S6. TVB-2640 and MβCD inhibit JEV replication**

(**A** and **B**) Cell viability was measured using CCK8 assays in HeLa cells treated with the TVB-2640 (**A**) and MβCD (**B**). (**C**) HeLa cells were incubated with JEV NJ2008 strain for 2 h (MOI = 0.5) and then treated with different concentrations of TVB-2640 for 24 h. JEV infection was analyzed by qRT-PCR. Data were expressed as the mean ± standard deviation (SD) of three independent experiments. (**D**) HeLa cells were incubated with JEV NJ2008 strain for 2 h (MOI = 0.5) and then treated with different concentrations of MβCD for 24 h. JEV infection was analyzed by western blotting. (**E**) Immunofluorescence analysis of JEV infection in DMSO, TVB-2640 (1 μM), or MβCD (1mM) treated HeLa cells. Scale bars, 20 µm.

**Figure S7.** **Knockdown of RRBP1 inhibits JEV replication**

(**A**) Western blotting of RRBP1 knockdown in WT HeLa cells. (**B**) JEV infection in siRRBP1 and siControl HeLa cells was determined using qRT-PCR. Data were expressed as the mean ± standard deviation (SD) of three independent experiments using a two-tailed t-test, ** *p* < 0.01.

**Figure S8. Model of the ER membrane concentration induced by NS1**

(**A**) JEV infection results in the concentration of ER membranes and the formation of MROs. (**B**) The enlarged image of MROs from panel A. (**C**) The enlarged view of a single RO from panel B. The images were created using BioRender.
